# A Survey of Map Construction and Management Methods for Single Embodied Agents

**DOI:** 10.3390/s26196242

**Published:** 2026-10-01

**Authors:** Chao Yan, Zeyang Feng, Fei Ye, Yang Sun, Yefei Gong, Yu Liu

**Affiliations:** 1 School of Electrical Engineering and Automation, Suzhou University of Technology, Suzhou 215500, China; 1250202004@szut.edu.cn (Z.F.); gong_yf@cslg.edu.cn (Y.G.); 2 Embodied Intelligent Industrial Robots and Human-Machine Collaboration Laboratory, Suzhou University of Technology, Suzhou 215500, China; 3 School of Instrument Science and Engineering, Southeast University, Nanjing 210096, China; yang_sun@seu.edu.cn; 4 School of Automobile Engineering, Suzhou University of Technology, Suzhou 215500, China; xyz.comyefei@outlook.com; 5 Key Laboratory of Quantitative Remote Sensing in Agriculture of Ministry of Agriculture and Rural Affairs, Information Technology Research Center, Beijing Academy of Agriculture and Forestry Sciences, Beijing 100097, China

**Keywords:** single embodied agent, map representation, map construction, map management, long-term autonomy

## Abstract

Embodied intelligent systems need to complete the closed loop of perception–understanding–decision–execution in the real world. One of their core capabilities is constructing, maintaining, and utilizing environmental maps. Maps not only support localization and navigation but also directly influence obstacle avoidance, safe interaction, mobile manipulation, long-term memory, and task planning. With the rapid development of multi-sensor fusion Simultaneous Localization and Mapping (SLAM), semantic understanding, and neural scene representations, robot maps have evolved from traditional two-dimensional occupancy grids to three-dimensional point clouds, voxel/octree, distance fields, mesh models, topological maps, object-level semantic maps, and dynamic scene graphs, further progressing toward continuous implicit maps centered on Neural Radiance Fields (NeRF), 3D Gaussian Splatting (3DGS), and open-vocabulary/foundation models. However, different map representations exhibit significant differences in geometric accuracy, queryability, updatability, computational/storage overhead, and support for downstream tasks. Meanwhile, long-running embodied systems also face engineering and theoretical challenges such as dynamic environments, sensor drift, multi-session fusion, version management, and forgetting/compression. This paper, oriented toward embodied intelligence applications, systematically reviews map representation forms, corresponding construction methods, and map management and update mechanisms. In terms of representation, a hierarchical classification of geometric/metric maps, semantic/structural maps, and neural/foundation model maps is presented, and their adaptability in navigation, manipulation, and language interaction is discussed. In terms of construction, traditional probabilistic mapping, geometric registration (ICP/NDT), graph optimization and factor graphs, semantic fusion and scene graph construction, as well as online optimization methods for neural implicit maps and 3DGS maps are systematically reviewed. In terms of management, key technologies such as submap management, map indexing and compression, dynamic environment updating, change detection, multi-session map fusion, and neural map continual learning and catastrophic forgetting mitigation are summarized. Finally, the paper concludes and envisions future development trends, emphasizing that hierarchical hybrid maps, task-driven cognition, and long-term continuous maintenance will be the key to deploying embodied intelligence in real-world settings.

## 1. Introduction

In recent years, with the rapid development of artificial intelligence, large models, multimodal perception, and robotics technologies, embodied intelligence has gradually become an important research direction for artificial intelligence progressing toward general intelligence [1]. Unlike traditional AI that relies on static data for perception or reasoning, embodied intelligence emphasizes agents continuously perceiving the environment through sensors, interacting with the real physical world via actuators, and completing autonomous learning and task execution within the closed-loop process of perception–understanding–decision–execution. As robotic applications gradually expand from structured environments to open scenarios such as home services, smart logistics, industrial manufacturing, and medical assistance, agents need to not only complete basic tasks such as localization and navigation but also to possess comprehensive capabilities including long-term autonomous operation, complex task planning, human–robot interaction, and environmental adaptation. In this process, maps have evolved from being merely the foundation for robot localization and path planning to becoming a unified environmental representation that integrates geometric structures, semantic information, spatial relationships, and even task knowledge, serving as critical infrastructure supporting embodied intelligence for long-term autonomous operation.

With the continuous maturation of multi-source sensor technologies such as LiDAR, RGB-D cameras, and visual–inertial systems and the integration of next-generation AI technologies such as deep learning, Neural Radiance Fields (NeRF), 3D Gaussian Splatting (3DGS), and foundation models into robot perception systems, the representation forms and construction methods of environmental maps are undergoing profound transformations. Map representations have evolved from early navigation-oriented 2D occupancy grid maps to 3D point cloud maps, voxel maps, distance field maps, mesh maps, topological maps, semantic maps, and further to neural implicit maps, 3DGS maps, and hybrid maps that deeply integrate heterogeneous geometric data such as triangular meshes and point clouds in structure [2]. Unlike traditional static maps, map systems for embodied intelligence need to provide not only high-precision spatial structures but also capabilities for dynamic environment understanding, multi-session map fusion, long-term information maintenance, continuous updating, and cross-task knowledge sharing. However, since different map representations exhibit significant differences in geometric accuracy, computational efficiency, semantic expressiveness, updating mechanisms, and task adaptability, there currently exists no single map representation that can simultaneously satisfy the diverse requirements of long-term embodied intelligent systems in localization, navigation, manipulation, interaction, and reasoning. Therefore, constructing a unified map system that balances geometric fidelity, semantic understanding, dynamic adaptability, and long-term maintainability has become an important direction in current embodied intelligence spatial cognition research.

In recent years, extensive research has been conducted on robot map representation, SLAM, 3D reconstruction, and semantic environment understanding, resulting in several representative survey works. Cadena et al. systematically summarized the development trajectory of visual SLAM, analyzing the evolution of robot spatial perception technologies from the perspectives of front-end perception, back-end optimization, loop closure detection, and long-term operation [3]. Campos et al., building on the development of ORB-SLAM3, systematically reviewed visual, visual–inertial, and multi-map SLAM systems, further reflecting the trend of modern SLAM systems toward multi-sensor fusion and long-term autonomous operation [4]. These works have laid important foundations for robot spatial localization and map construction but primarily focus on state estimation, optimization frameworks, and SLAM system design, with relatively limited coverage of map representation forms, environmental knowledge expression, and map lifecycle management.

As the focus of robotics research gradually shifts from spatial localization to environmental understanding and task execution, researchers have begun to approach 3D spatial representation and semantic environment modeling from a more systematic perspective. Rosen et al. [5] systematically reviewed 3D maps, semantic mapping, dynamic scene graphs, and long-term robot environment modeling centered on spatial perception technologies, providing new research perspectives for the development of embodied intelligence environmental representations. Subsequently, related research further expanded from single geometric modeling to multi-level spatial representation systems integrating visual, semantic, and knowledge reasoning capabilities. In recent years, several survey works have systematically analyzed point cloud, voxel, distance field, mesh, scene graph, neural implicit representation, and 3D Gaussian representation technologies, pointing out that future robot maps will gradually evolve from single geometric representations toward unified spatial representations integrating geometric structures, semantic knowledge, and task information [6,7]. Furthermore, with the development of foundation models and embodied intelligence technologies, the latest research focuses more on the continual learning, semantic knowledge acquisition, and dynamic world modeling capabilities of robot environmental models, emphasizing that future agents need to construct long-term environmental representations supporting perception, reasoning, prediction, and interaction [8,9]. Although these surveys have greatly advanced the understanding of SLAM systems, 3D scene representations, and semantic mapping, they share three common limitations. First, they largely treat map representation, map construction, and map management as separate topics, so the intrinsic dependency among them—a representation dictates how it is built and how it can be efficiently updated—is rarely analyzed end-to-end. Second, most existing surveys are organized around a single map category (e.g., neural implicit or 3DGS) or a single SLAM system and thus cannot guide the practitioner who must choose among geometric, semantic, and neural representations for a concrete embodied task. Third, and most importantly, the lifecycle management of maps for a single agent operating over long time horizons—dynamic-environment updating, multi-session fusion, map compression, continual learning, and forgetting—is either mentioned only in passing or restricted to multi-robot/cloud settings. As summarized in Table 1, no existing survey unifies these three threads from the perspective of a single embodied agent that must autonomously maintain its own maps with bounded onboard resources.

To address these gaps, this paper presents a systematic survey of map construction and management technologies for embodied intelligence, organized along the technical thread of map representation-map construction-map management. First, we systematically review typical map representations including 2D grid maps, 3D voxel maps, distance field maps, feature maps, mesh maps, topological maps, semantic maps, neural implicit maps, 3DGS maps, and hybrid maps, summarizing their development histories, mathematical models, technical characteristics, and applications in embodied intelligence. Second, following the development trajectory of SLAM technologies, we analyze the construction methods corresponding to different map representations, emphasizing the evolution of key technologies such as probabilistic mapping, graph optimization, semantic fusion, and neural field optimization. Finally, targeting single-agent long-term autonomous operation scenarios, we systematically summarize the development of map management and updating mechanisms, focusing on the trends in incremental map updating, dynamic environment mapping, multi-session fusion, map compression, continual learning, and neural map online updating, providing theoretical references and technical insights for constructing long-term autonomous map systems for embodied intelligence.

Compared with existing surveys, the main contributions of this work are fourfold:

**Lifecycle-centric organization.** We are, to the best of our knowledge, the first survey to analyze map representation, map construction, and map management as one coupled chain for a single embodied agent, explicitly separating one-shot map building (Section 3) from lifelong map maintenance (Section 4).

**A principled three-category taxonomy.** Instead of an enumeration of representations, we organize ten map forms into geometric/metric, semantic/structural, and neural/foundation-model maps, with the classification criteria stated explicitly.

**Task- and resource-oriented quantification.** We consolidate a quantitative comparison of memory overhead, update latency, geometric accuracy, computational cost, and adaptability to manipulation and language-guided navigation tasks.

**Deployment-oriented analysis**. We add a dedicated discussion of hardware–software co-design and edge deployment trade-offs for neural implicit and 3DGS maps (Section 4.3), and decompose the future outlook into concrete open research sub-problems (Section 5.2).

This paper is organized into five Sections as follows: Section 1 is the introduction, presenting the research background, research content, and main contributions. Section 2 surveys spatial representation and environmental mapping technologies for embodied intelligence, analyzing the development history, mathematical models, and application characteristics of various map representations. Section 3 summarizes the construction methods corresponding to different map representations and the evolution of key technologies. Section 4 focuses on map management and updating mechanisms for single agents under long-term operation conditions. Section 5 concludes the paper and envisions future directions.

## 2. Spatial Representation and Environmental Mapping Technologies for Embodied Intelligence

In the interdisciplinary research domain of mobile robotics and Embodied AI, spatial representation and mapping constitute the foundational cornerstone for agent perception, decision-making, and physical interaction. The rise of embodied intelligence marks a fundamental shift in artificial intelligence from traditional disembodied data processing toward continuous closed-loop interaction with the physical world. In this paradigm, the system is required not only to answer ‘where am I’ (state estimation) and ‘what is around me’ (geometric mapping), but also to deeply understand ‘what can I do’ (physical affordance) and ‘how to respond to natural language instructions’ (semantic and command anchoring).

**Figure 1:**
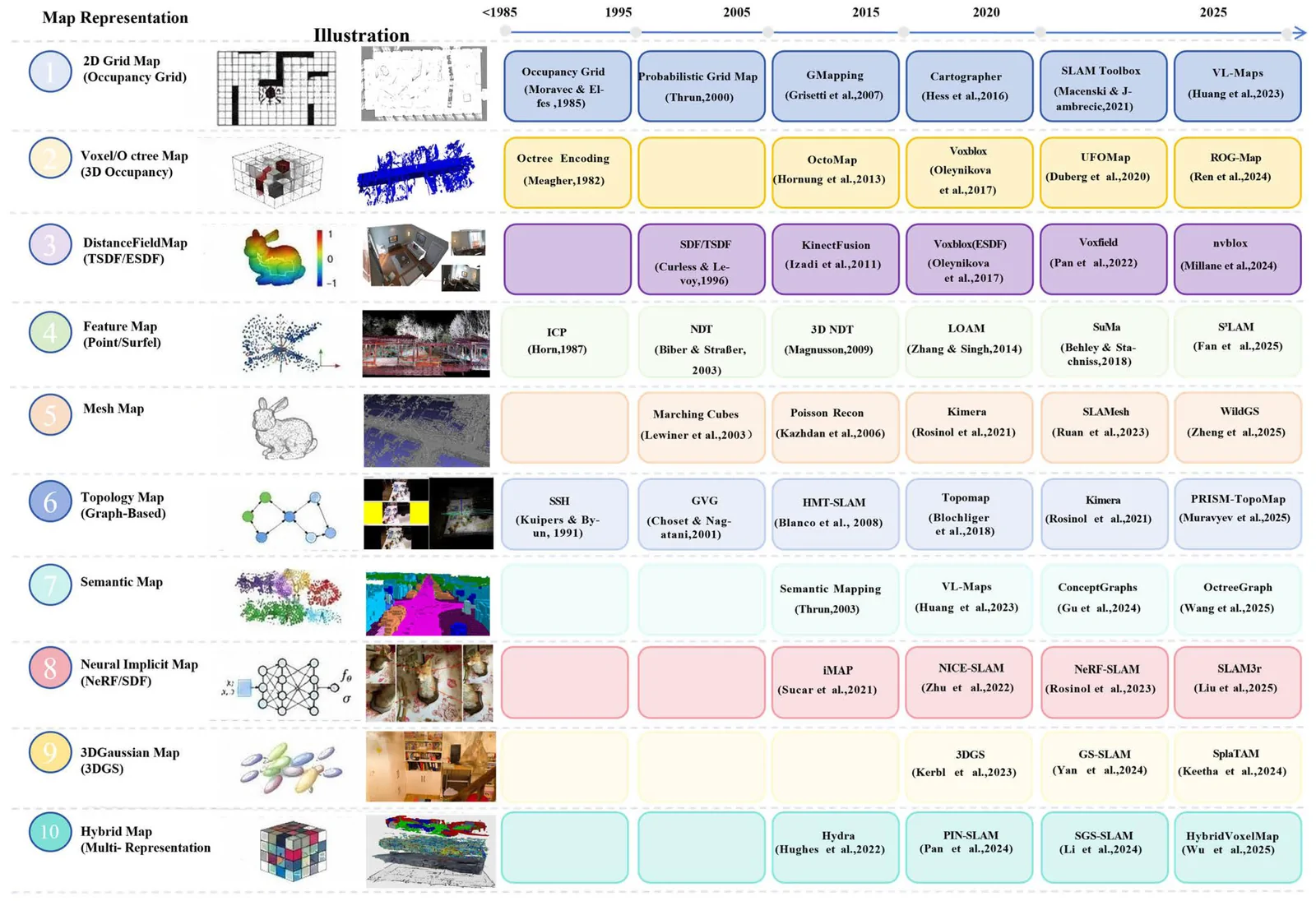
Map development timeline. References for the works shown, grouped by category: 2D occupancy grids [10,11,12,13,14,15]; Voxel and octree maps [16,17,18,19,20]; Distance fields [17,21,22,23,24]; Feature maps [25,26,27,28,29,30]; Mesh maps [31,32,33,34,35]; Topological maps [32,36,37,38,39,40]; Semantic maps [15,41,42,43]; Neural implicit maps [44,45,46,47]; 3D Gaussian maps [48,49,50]; Hybrid maps [51,52,53,54].

From early 2D probabilistic grid maps to 3D dense reconstruction, and now to open-vocabulary scene graphs integrated with large multimodal foundation models and continuous neural implicit radiance fields, the connotation and data structures of maps have undergone profound evolution, as shown in Figure 1. This survey provides an exhaustive technical analysis of mainstream map representations within the context of embodied intelligence, strictly following the trajectory of technological evolution, covering their theoretical origins, mathematical foundations, core algorithmic architectures, and practical adaptability across different task flows.

We classify the ten map forms into three categories according to how the environment is encoded: geometric/metric maps store geometry explicitly; semantic/structural maps add object identity and relational structure; neural/foundation-model maps encode the scene implicitly through learnable parameters. The defining criterion of each category is summarized in Table 2.

### 2.1. Two-Dimensional Occupancy Grid Maps

The 2D Occupancy Grid Map is one of the most classical methods for robot environment representation. Early robots relied on sensors such as sonar for environmental perception, but due to observation noise and uncertainty, traditional geometric maps could not accurately describe real environments. Moravec and Elfes proposed the occupancy grid map in 1985, discretizing environmental space into regular grids and describing the occupancy state of each region using probabilistic representations [10]. Subsequently, Thrun further refined probabilistic mapping theory, introducing Bayesian estimation into the map updating process, making 2D grids a foundational representation method in SLAM and autonomous navigation systems [11]. 2D grid maps typically use 2D arrays to store spatial states, with each grid cell representing the probability of obstacle existence through occupancy probability. Its core objective is to estimate the environmental state under sensor observation and robot pose constraints. For the *i*-th grid cell, its occupancy probability can be expressed as

*p*(*m_i_*|*z*_1:*t*_, *x*_1:*t*_) (1)

where *m_i_* denotes the occupancy state of the *i* cell, *z*_1_:*t* denotes the robot’s sensor observations from time 1 to t, and *x*_1_:*t* denotes the robot’s historical poses. Since directly computing the joint probability distribution of the entire environment would lead to enormous computational complexity, Moravec and Elfes introduced the zero-order Markov Random Field (MRF) assumption, considering each grid cell independent of others. In engineering implementation, to avoid numerical instability from probability multiplication, the Log-odds form is typically used:

(2) $$L(m_{i})=\log\left(\frac{p(m_{i})}{1-p(m_{i})}\right)$$

With Recursive Bayesian Update, the grid state can be updated incrementally:

*L*(*m_i_*|*z*_1:*t*_) = *L*(*m_i_*|*z*_1:*t*−1_) + *L*(*m_i_*|*z_t_*) − *L*(*m_i_*) (3)

where *L*(*m_i_*|*z_t_*) is obtained through the Inverse Sensor Model by converting single wide-beam observations into probability increments for each grid. Since the prior probability *p*(*m_i_*) = 0.5 is typically assumed, the prior Log-odds is 0, and the update process can be simplified to the accumulation of historical states and current observations, achieving efficient incremental mapping [10,11].

### 2.2. Three-Dimensional Voxel Maps

As robotic applications expanded to UAV exploration, complex terrain locomotion, and 3D manipulation, 2D grids lacking height information cannot describe spatial structures such as stairs and overhanging obstacles, driving the development of 3D voxel representations. While dense voxel grids allow direct modeling, they face severe memory and computational pressure as scale and resolution increase. To address this, Hornung et al. proposed the OctoMap probabilistic octree mapping framework, which adaptively adjusts resolution through octree structures—using large nodes in open areas and recursively subdividing in geometrically detailed regions—combined with probabilistic occupancy mechanisms to explicitly represent occupied, free, and unknown regions, significantly reducing storage overhead [16]. As a foundational spatial perception layer for embodied intelligence, OctoMap can provide environmental constraints for obstacle avoidance, terrain analysis, and navigation. During map updating, OctoMap performs probabilistic updates on traversed voxels along sensor observation rays (ray-casting), with Log-odds form at its core. For node n, the update formula is:

*L*(*n*|*z*_1_:*t*) = *max*(*min*(*L*(*n*|*z*_1_:*t* − 1) + *L*(*n*|*z_t_*), *l_m_ax*), *l_m_in*) (4)

where n represents an octree node, and l_max and l_min are the upper and lower Log-odds limits. The Clamping Policy prevents the over-confidence problem caused by long-term observations, enabling the map to respond more quickly to obstacle changes in dynamic environments [16]. Additionally, OctoMap introduces a dynamic Node Pruning strategy: when eight child nodes share the same occupancy state, they are merged into their parent node without loss, significantly reducing memory requirements, making it one of the most representative methods in robot 3D occupancy mapping.

However, 3D voxel maps remain discrete spatial representations, primarily describing ‘whether space is occupied’ without directly providing continuous distance relationships between robots and obstacles. Therefore, to meet the needs of high-speed motion planning and fine control, research has further developed Distance Field Maps based on continuous distance representation.

### 2.3. Distance Field Maps (TSDF/ESDF)

Although 3D voxel maps effectively describe spatial occupancy, their discrete representations cannot directly provide continuous distance information and are prone to staircase effects, limiting their application in trajectory optimization and fine manipulation. To address this, researchers proposed Distance Field Maps that describe the distance relationship between space and obstacles through continuous functions. Early Truncated Signed Distance Functions (TSDF) achieve continuous smooth surface recovery by storing the signed distance from spatial points to surfaces, but they only focus on narrow-band regions near obstacle surfaces, lacking distance description for free space [21]. In contrast, Euclidean Signed Distance Fields (ESDFs) compute the Euclidean distance from any spatial position to the nearest obstacle surface, with the Voxblox framework achieving real-time incremental ESDF computation, substantially improving mapping efficiency [17]. In embodied intelligence tasks, ESDFs provide continuous distance gradients, supporting gradient-based trajectory optimization algorithms such as CHOMP and TrajOpt to compute collision costs and guide robot obstacle avoidance, thereby enabling smoother and safer motion control [55].

In the TSDF construction process, for each voxel center *x* and sensor depth observation point *p* (with sensor center s), the projected physical distance d is computed as:

*d*(*x*, *p*, *s*) = ‖*p* − *x*‖⋅*sign*((*p* − *x*)⋅(*p* − *s*)) (5)

Subsequently, the system recursively fuses historical distance information using weighted averaging based on observation confidence weight W:

(6) $$D_{i+1}(x,p,s)=\frac{W_{i}(x)D_{i}(x)+w(x,p)d(x,p,s)}{W_{i}(x)+w(x,p)}$$

where the update range is typically limited within a truncation distance delta near the surface to reduce the influence of far-field depth noise [21]. Compared to TSDFs, which employ local distance fusion, ESDFs focus on solving the global distance propagation problem. Voxblox achieves incremental distance field updates through the Wavefront Propagation mechanism:

(7) $$d_{\mathrm{ESDF}}(x)=\underset{y\in N(x)}{\min}\left(d_{\mathrm{ESDF}}(y)+\|x-y\|\right)$$

When the environment changes, the system maintains Raise Queue and Lower Queue separately: when new obstacles appear, the Lower Queue rapidly propagates new distance constraints; when obstacles are removed, the Raise Queue clears invalid distance information, thereby achieving real-time updates in dynamic environments [17].

Overall, Distance Field Maps break through the limitation of voxel maps that only express occupancy status, transforming discrete space into continuous geometric constraints, providing a more optimization-friendly environmental representation for robot safety planning, dynamic obstacle avoidance, and fine manipulation. However, they mainly focus on geometric distance relationships, still lacking expressive capability for object textures, semantic categories, and complex appearance information, thus further driving the development of higher-level representations such as point clouds, Surfels, and semantic maps.

### 2.4. Feature Maps (Point Cloud and Surfel Maps)

While distance field maps provide continuous spatial constraints, their expressive capability for object surface textures and local fine structures is limited, making them insufficient for high-precision interaction tasks such as robotic arm grasping and visual servoing. To address this, research further developed high-precision surface map representations such as Point Cloud and Surfel maps. Point cloud maps directly use sensor sampling points to describe surfaces; although they avoid fixed resolution limitations and preserve original information, their unordered nature, data sparsity, and lack of explicit topological structure make it difficult to directly support high-precision surface modeling. Surfel maps, by modeling local surfaces as tiny patches with position, normal vector, color, and weight (such as the ElasticFusion system [56]), bridge the gap between discrete point clouds and continuous surfaces, offering better continuity and visual consistency [25,57,58,59]. In embodied intelligence systems, point cloud processing frameworks provide efficient tools for handling point clouds and extracting local geometric features, thereby sup-porting 3D perception and downstream geometric processing [60].

In the ElasticFusion framework, each Surfel primitive not only contains spatial position *v_k_* and surface normal *n_k_*, but also maintains appearance color, weight confidence *w_k_*, and timestamp attributes [56]. When new depth observations successfully match existing Surfels, the system performs incremental fusion based on observation confidence *α*:

(8) $${\bar{v}}_{k}\leftarrow \frac{{\bar{w}}_{k}{\bar{v}}_{k}+\alpha v_{i}}{{\bar{w}}_{k}+\alpha }$$

(9) $${\bar{n}}_{k}\leftarrow \frac{{\bar{w}}_{k}{\bar{n}}_{k}+\alpha n_{i}}{{\bar{w}}_{k}+\alpha }$$

(10) $${\bar{w}}_{k}\leftarrow {\bar{w}}_{k}+\alpha$$

where *v_i_* and *n_i_* represent the position and normal of the current observation point respectively, achieving continuous surface model updating through weighted fusion [57]. Unlike traditional pose-graph optimization methods, ElasticFusion employs Non-rigid Deformation Graph for global correction after loop closure detection. The system establishes deformation nodes through spatial distance and temporal information, and uses a smooth deformation model to synchronously adjust the positions and normals of a large number of Surfels, thereby avoiding the computational overhead of large-scale re-optimization and achieving map consistency maintenance under long-term operation [59].

However, point cloud and Surfel maps remain discrete surface sampling representations. Although they preserve rich geometric details, they lack explicit surface topological structure, presenting limitations in tasks such as collision detection, physical simulation, and contact force computation. Therefore, further research began utilizing triangular mesh structures to construct continuous surface models, namely Mesh Maps.

### 2.5. Mesh Maps (Triangular Mesh and Elevation Maps)

As robotic tasks expand to physical interaction and complex manipulation, Surfel maps in discrete sampling form, lacking explicit topological connections, face limitations in collision detection, dynamic simulation, and contact force computation, driving the development of Triangle Mesh-based and Elevation Map representations. Mesh maps construct continuous surface topological structures through vertices, edges, and triangular facets, typically extracted from TSDF via the Marching Cubes algorithm [31,60,61]. They avoid the staircase effect of voxels while fusing geometric and visual information through explicit topological structure and texture mapping, and are widely applied in simulation, digital twins, and fine manipulation. For unstructured terrain encountered by ground and legged robots, 2.5D elevation maps provide effective support by storing height values and uncertainty for each grid cell, integrating attributes such as height, normal vectors, and roughness [62,63,64,65]. In embodied intelligence tasks, Meshes can directly serve as collision models in physics engines for dynamic computation, while elevation maps sacrifice partial spatial expression in exchange for extremely high computational efficiency, providing traversability assessment for fine foothold planning and large-scale outdoor locomotion for legged robots.

The core of Mesh construction is extracting the Zero Isosurface from the implicit distance field. In the Marching Cubes algorithm, the system traverses eight corner points of a voxel and determines whether the surface passes through the current voxel based on sign changes, computing triangle vertex positions through linear interpolation [31]:

(11) $$\mu =\frac{\mathrm{iso}-v_{1}}{v_{2}-v_{1}}$$

*p* = *p*_1_ + *μ*(*p*_2_ − *p*_1_) (12)

Subsequently, corresponding triangular patches are generated based on a predefined topology lookup table, achieving continuous surface reconstruction. For 2.5D elevation maps, single height observations may contain considerable uncer-tainty owing to pose drift and sensor noise. Recursive estimation methods such as Kalman filtering can therefore be used to fuse height observations over time [66], as follows:

(13) $${\hat{h}}_{\mathrm{new}}=\frac{\sigma_{z}^{2}\hat{h}+\sigma_{h}^{2}z}{\sigma_{h}^{2}+\sigma_{z}^{2}}$$

where $\hat{h}$ is the historical height estimate, *z* is the current observation height, and $\sigma_{z}^{2}$ and $\sigma_{h}^{2}$ represent historical estimation and measurement noise variances, respectively. Through continuous fusion of multi-frame observations, elevation maps can maintain stable height estimation in complex terrain environments, providing reliable basis for robot motion control [66,67].

### 2.6. Topological Maps

As robotic application scenarios expand to open and large-scale spaces, relying solely on high-precision metric maps gradually exposes problems such as global coordinate drift, dramatic increases in storage and computation, and difficulty in understanding human spatial concepts like rooms and corridors. To address this, researchers proposed Topological Maps, which abstract environments into places and reachability relationships, sacrificing partial geometric details in exchange for stronger scalability and long-term stability. Modern spatial perception systems such as Kimera represent environments through hierarchical structures that integrate metric, semantic, and topological information, while Kimera-Multi further extends this representation to collaborative multi-robot mapping [32,68,69]. In these representations, the topological layer describes the environment using nodes and their connectivity relationships. In terms of representation, a topological map typically consists of place nodes (Nodes/Places) and connecting edges (Edges). Nodes represent spatially meaningful positions such as rooms, corridor intersections, or key landmarks; edges represent reachability relationships between different places, with weights that can describe path costs, action constraints, or connection probabilities. In mathematical representation, a topological map can be expressed as a graph structure:

G = <V, E >(14)

where V denotes the set of place nodes and E denotes the connection relationships between nodes. In a graph-based topological representation, the robot robot’s continuous motion pro-cess can be further abstracted into discrete state transitions:

P(*s_t_*_+1_|*s_t_*, *a_t_*) (15)

where *s_t_* represents the current spatial state and *a_t_* represents the robot’s executed action. Compared to geometric navigation methods that rely on precise coordinates, topological navigation mainly focuses on connection relationships between states, thereby reducing the impact of accumulated drift on long-term task execution [36,70].

In embodied intelligence systems, topological maps provide efficient global task planning capability, enabling robots to quickly locate target areas through a global-topology-plus-local-geometric-control architecture and complete navigation by combining local metric maps, making them an important design for long-term autonomous robots. However, since topological maps discard precise geometric information, they cannot directly support low-level control tasks such as obstacle avoidance and grasping. Therefore, hybrid map systems combining topological representations with metric maps and semantic information have become an inevitable trend for balancing spatial structure and environmental understanding.

### 2.7. Semantic Maps

Although metric maps can precisely describe geometric structures, they exhibit a semantic gap due to the lack of understanding of environmental content and functional attributes, limiting complex human–robot interaction and high-level task planning. To address this, researchers have integrated object categories, instance information, and semantic attributes into geometric maps, constructing Semantic Maps or metric-semantic hybrid maps, among which frameworks such as Kimera-Semantics have successfully achieved real-time 3D semantic reconstruction [32,68,69]. Current semantic maps are mainly divided into two categories: dense semantic voxel/grid maps maintain category probabilities within voxels to fully describe semantic distributions, but incur significant storage and computation overhead; object-level maps abstract space into independent entity nodes containing category, 3D bounding box, and position through object detection and instance segmentation, possessing higher abstraction capability and being more suitable for object retrieval and task planning. In embodied intelligence applications, semantic maps can not only optimize obstacle avoidance strategies by assigning traversal costs to different categories, but also transform complex mobile manipulation into object relationship reasoning, thereby significantly reducing planning complexity.

In semantic mapping frameworks such as Kimera-Semantics, multi-view semantic fusion typically employs probability-based voxel update methods. For spatial voxel *v*, the system maintains its probability distribution belonging to different semantic categories *c* [32]:

P(*l_v_* = *c*|*z*_1:*t*_) = *η*⋅P(*z_t_*|*l_v_* = *c*)⋅P(*l_v_* = *c*|*z*_1:*t*−1_) (16)

where *z_t_* represents the current image semantic observation and *η* is the normalization coefficient. Observation probabilities typically originate from the output results of 2D semantic segmentation networks (such as Mask R-CNN, DeepLab, etc.). Through the continuous fusion of multi-frame and multi-view observations, the system can reduce the effects of occlusion and individual-frame misclassification, obtaining more stable 3D semantic representations [32,68].

Overall, semantic maps break through the limitation of traditional geometric maps that can only describe ‘where is the space,’ enabling robots to further possess the cognitive capability of ‘what is in the space.’ However, semantic maps based on discrete voxels and object labels still face limitations in storage scale, open-category recognition, and long-term update capability. Therefore, in recent years, research has further explored Neural Implicit Maps that use neural networks to continuously represent environments.

### 2.8. Neural Implicit Maps

Traditional maps rely on explicit structures such as grids, voxels, point clouds, or meshes, constrained by fixed resolution, storage scale, and discretization errors, making it difficult to meet the continuous surface expression and fine manipulation requirements of complex scenes. To address this, researchers have explored Neural Implicit Maps that use neural networks to directly learn continuous spatial mappings. This method encodes geometric structures and appearance into network parameters and spatial features, breaking through resolution limitations and naturally expressing continuous surfaces and view-dependent appearance. To address the issues of slow convergence and insufficient detail when using early MLPs to fit scene functions, Zhu et al. proposed NICE-SLAM, which combines hierarchical feature grids with lightweight MLP decoders to predict occupancy and color for dense mapping and camera tracking [44]. Related developments include Sucar et al.’s iMAP for online implicit mapping and positioning [45], Müller et al.’s multiresolution hash encoding for efficient neural representations [71], and Mildenhall et al.’s NeRF for novel-view synthesis [72]. Differentiable rendering further enables gradient-based optimization of scene representations and camera poses in neural implicit SLAM [44].

In the NICE-SLAM framework, 3D scenes are represented as feature grids containing coarse, mid, and fine scales. For a spatial query point *p*, the system obtains multi-scale features through trilinear interpolation and inputs them into a lightweight MLP network to predict the corresponding implicit geometric properties (such as SDF) and color information [44]. Its mapping and tracking process is essentially an alternating optimization process based on rendering errors [44,45,71]. The system constructs a joint photometric and depth loss function by comparing real observation images with neural rendering results:

(17) $$L=\sum_{r\in R}(\|C(r)-\hat{C}(r){\|}_{1}+\lambda \|D(r)-\hat{D}(r){\|}_{1})$$

where *C*(*r*) and *D*(*r*) represent real observed color and depth, and $\hat{C}(r)$ and $\hat{D}(r)$ represent neural rendering predictions. Through gradient descent optimization, this loss function is used to simultaneously update the map representation and camera pose. In the mapping phase, the system fixes the camera pose and only optimizes the feature grid and MLP parameters, causing the implicit field to gradually approach the real environment; in the tracking phase, the map parameters are frozen, and the camera pose is optimized through rendering error backpropagation, achieving high-precision localization [44].

However, neural implicit maps still face issues such as large online optimization computation, insufficient real-time performance, and limited dynamic environment adaptation capability. Therefore, to further improve real-time mapping capability in robotic systems, research has further developed 3D Gaussian Splatting maps based on explicit continuous representation.

### 2.9. 3D Gaussian Splatting (3DGS) Maps

Although neural implicit maps significantly improve 3D reconstruction quality, their MLP-dependent ray sampling and volume rendering computations are complex, making it difficult to meet robot real-time localization and online mapping requirements. To address this, 3DGS was proposed and rapidly became a research hotspot [48,73,74]. Unlike NeRF’s implicit expression, 3DGS adopts an explicit continuous representation, abstracting the environment into a large number of learnable 3D Gaussian primitives (ellipsoids) containing spatial position, scale, orientation, color, and transparency (covariance and parameters), combining the advantages of point cloud efficient updating and neural rendering high-quality visual expression, and leveraging GPU parallel computing and GS-SLAM frameworks to achieve high-frame-rate online incremental mapping [49,50,73]. During optimization, Gaussian primitives can adaptively adjust, split, and delete based on observation errors, thereby precisely fitting real object surfaces and breaking through fixed resolution limitations to recover fine structures, complex textures, and highly reflective high-frequency details [50,73]. In embodied intelligence applications, the efficient rendering and optimization capabilities of 3DGS can support realistic environmental reconstruction, localization, and interactive perception under challenging visual conditions [75,76].

In mathematical representation, each 3D Gaussian primitive is defined by center position mu, covariance matrix Sigma, color parameters *c*, and transparency alpha, with its spatial influence function:

(18) $$G(p)=\exp\left(-\frac{1}{2}{\left(p-\mu \right)}^{T}\Sigma^{-1}(p-\mu )\right)$$

where the covariance matrix determines the spatial shape of the Gaussian ellipsoid. To ensure the covariance matrix remains positive definite during optimization, it is typically decomposed into rotation matrix **R** and scale matrix **S**:

**Σ** = **RSS**^T^R^T^ (19)

In the rendering stage, 3DGS differs from NeRF’s volume integration method, employing GPU-optimized differentiable rasterization: first, 3D Gaussians are transformed to the 2D image plane through camera projection:

**Σ**_2*D*_ = **JWΣ**_3*D*_**W**^T^**J**^T^ (20)

where **W** is the view transformation matrix containing camera extrinsic parameters. Subsequently, the Gaussian primitives covering pixels are sorted by depth, and the final color is calculated through *α* blending:

(21) $$C=\sum_{i\in N}c_{i}\alpha_{i}\prod_{j=1}^{i-1}(1-\alpha_{j})$$

Since the entire rendering process only involves matrix operations and explicit parameter optimization without complex neural network inference, it achieves high-speed rendering while directly propagating error gradients to Gaussian parameters, achieving simultaneous localization and map optimization [76,77,78,79,80]. However, 3DGS still faces challenges in dynamic environment processing, semantic understanding, and large-scale map management; since its optimization objective mainly focuses on visual reconstruction quality, its expressive capability for occupancy status, safety distances, and semantic relationships required for robot navigation is limited. Therefore, future embodied intelligence systems are more inclined to adopt hybrid map systems that integrate 3DGS with voxels, semantic graphs, and topological structures.

### 2.10. Hybrid Maps

Through the development of the aforementioned 2D grids, 3D voxels, distance fields, surface models, topological structures, semantic maps, and neural implicit representations, it becomes evident that a single map representation can hardly simultaneously satisfy the multi-scale, multi-task requirements of embodied intelligence systems in long-term operation. For example, local obstacle avoidance and motion control require continuous distance constraints (such as ESDF); large-scale path planning relies on highly abstract, highly compressed topological structures; environmental understanding and human–robot interaction require fused semantic information; and high-fidelity simulation and visual interaction tasks require high-quality 3D appearance representations. Therefore, in recent years, robotics research has gradually shifted from single map representations to multi-layer fused Hybrid Map architectures.

The core idea of hybrid maps is to associate and fuse map representations at different abstraction levels within a unified spatial coordinate system, forming a robot internal World Model that integrates geometric, semantic, and task reasoning capabilities. It is not simply a superposition of multiple data structures, but rather information complementarity through cross-layer constraints: the bottom geometric layer is responsible for precise spatial localization and collision constraints, the middle semantic layer describes environmental entities and category relationships, and the top topological layer supports large-scale task planning. For example, Tian et al. proposed Kimera-Multi system, in a multi-robot collaborative mapping framework, fuses sparse visual odometry, dense 3D meshes, and semantic scene representations, achieving consistency maintenance of multi-robot environmental cognition [69].

Structurally, a typical hybrid map usually employs a layered representation:

M = {M_geo_, M_sem_, M_topo_, M_neural_} (22)

where M_geo_ represents the geometric metric layer (such as Voxel, ESDF, Mesh, or 3DGS) for precise spatial constraints; M_sem_ represents the semantic layer for describing object categories, instance relationships, and open-vocabulary information; M_topo_ represents the topological layer for supporting large-scale spatial reasoning and task planning; M_neural_ utilizes neural implicit fields or 3DGS to provide continuous, high-fidelity environmental appearance representation. In practical robot systems, different map layers are dynamically invoked according to task requirements. For global navigation tasks, the system typically performs fast path search in the topology layer to reduce computational overhead in large-scale environments; when the robot enters the target area and performs fine manipulation, it switches to the local geometry layer, providing millimeter-level collision constraints through occupancy grids, ESDF, or Mesh. Meanwhile, the semantic layer continuously provides object categories and spatial relationship information for task planning, achieving the transformation from spatial localization to environmental understanding [69].

**Figure 2:**
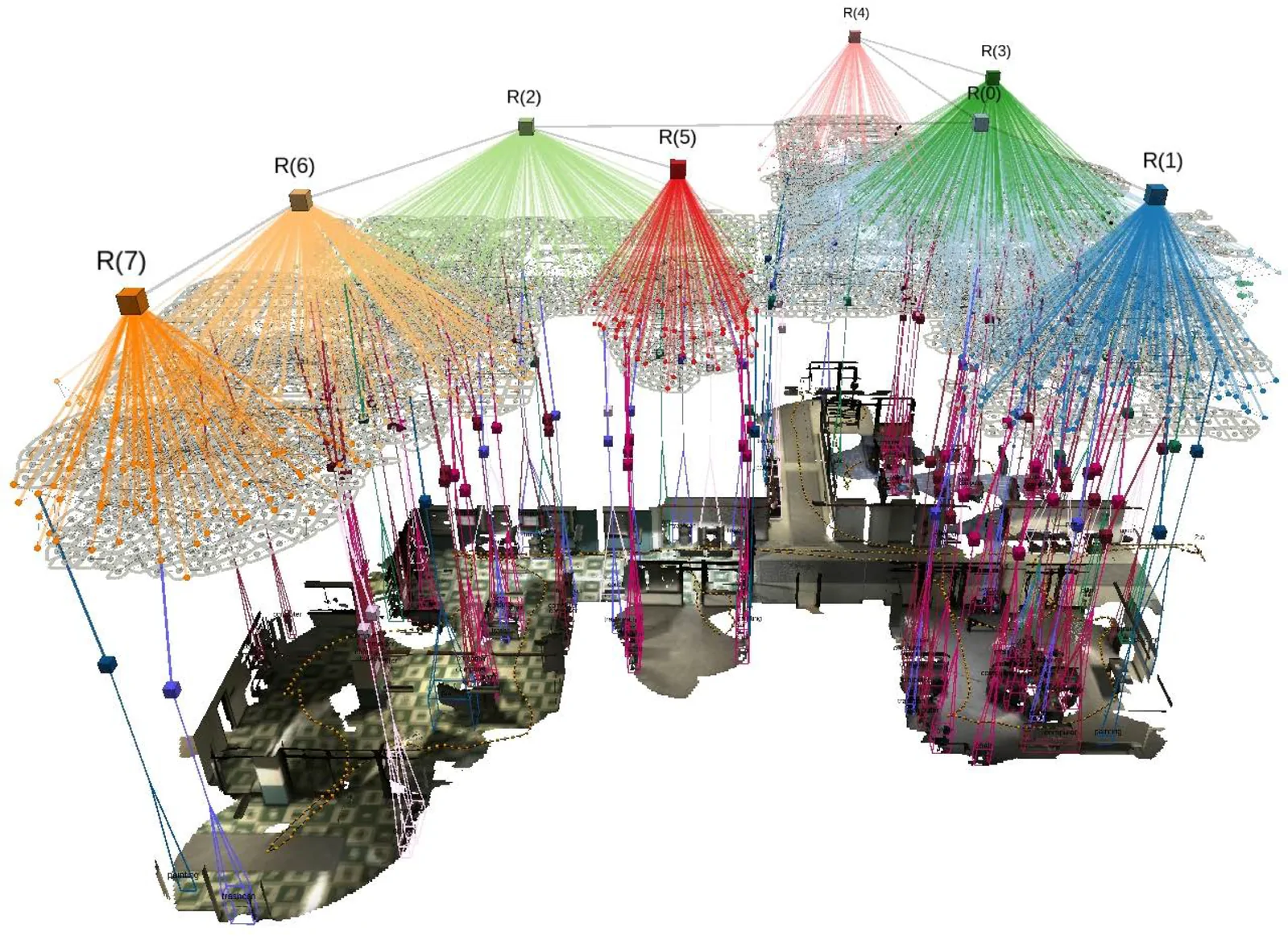
Hybrid map [54].

As shown in Figure 2, typical hybrid maps tightly connect spatial geometric details with topological and semantic relationships through layered or multi-module coupling. The core mathematical problem of hybrid maps is not simple data fusion but rather joint optimization under different modal constraints. The robot state can typically be represented as a Maximum A Posteriori (MAP) problem containing pose variables *X*, geometric metric layer M_geo_, semantic layer M_sem_, and topological structure layer M_topo_ [81]:

E_total_(*X*, M_geo_, M_sem_, M_topo_) = E_SLAM_(*X*) + E_dense_(*X*, M_geo_) + E_fusion_(M_geo_, M_sem_) + E_struct_(M_geo_, M_topo_) (23)

where E_SLAM_ represents pose estimation error, E_dense_ represents dense geometric reconstruction constraints, E_fusion_ represents geometric-semantic association error, and E_struct_ represents topological structure consistency constraints. Through Factor Graph optimization, information at different levels can be mutually corrected within a unified framework [81].

In engineering implementation, modern hybrid map systems (such as S-Graphs+ and Kimera-Multi [69,82]) typically employ multi-threaded asynchronous architectures: the front-end Tracking thread utilizes sparse visual features for high-frequency pose estimation to ensure localization stability; the back-end mapping thread updates dense geometric maps such as Mesh, Voxel, or neural representations based on stable trajectories; the semantic and topological modules run at lower frequency, continuously updating environmental structures and object relationships. Loop-closure correction, non-rigid deformation, and cross-layer consistency maintenance are lifecycle concerns and are discussed in detail in Section 4.2. Overall, hybrid maps represent the development trend of robot map representations from single spatial models to multi-layer World Models. Future embodied intelligence systems will further integrate geometry, semantics, topology, and neural representations, going beyond physical descriptions of ‘where’ and ‘what’ in the environment to comprehensively support robot high-level understanding, environmental prediction, and autonomous decision-making.

As shown in Table 3, modern embodied intelligence hybrid map systems present clear dimensional deconstruction and technical complementarity characteristics based on core data flow elements and application scenarios: Topometric hybrid maps combine local geometry with global topology to effectively mitigate large-scale accumulated drift and achieve millisecond-level pathfinding and are widely applied in warehouse logistics and shopping mall long-range navigation. Sparse–dense surface hybrid architectures decouple high-frequency tracking from low-frequency mapping computation, providing rigid body interfaces usable for friction simulation for dexterous manipulation. 2–2.5D elevation hybrid architectures evaluate uneven terrain under low computational power, extracting slope normals and roughness to support dynamic foothold planning for quadruped robots in field environments. Object-relation language graph hybrid architectures integrate instance point clouds with open-vocabulary features (such as CLIP/LLM), supporting commonsense-based natural language reasoning and open-world object search. Explicit-implicit neural raster hybrid architectures combine explicit spatial indexing with NeRF/3DGS, greatly accelerating ray queries and recovering transparent or highly reflective materials, providing differentiable rendering engines for high-fidelity reconstruction and digital twins in complex environments.

It would be misleading to present hybrid maps only by their advantages. Hybrid architectures pay concrete costs that a single representation does not: cross-representation consistency maintenance (a loop-closure correction must be propagated to every layer without contradiction), higher engineering complexity (multi-threaded asynchronous front/back-ends must synchronize at different rates), and greater total memory and compute (each layer carries its own bookkeeping). The decision should therefore be task-driven rather than defaulting to hybrid: a single explicit map suffices—and is more robust—when the task is navigation in a bounded, structured environment, while a hybrid map is justified only when the task genuinely spans multiple abstraction levels at once (global planning, safe local control, object manipulation, high-fidelity interaction). The value of a hybrid map is proportional to the number of distinct task demands the agent must satisfy simultaneously.

### 2.11. Concluding Remarks on Spatial Representation and Mapping

Based on the theoretical characteristics and engineering limitations of various map representations, the selection of spatial maps in embodied intelligence system architectures essentially needs to balance three aspects: task, hardware, and long-term operation. In large-scale navigation scenarios involving cross-region movement and high-speed driving, 2D occupancy grids, 3D OctoMap, or ESDF continuous distance fields are currently the most cost-effective choices in terms of computation, memory overhead, and system robustness. For precision physical operations involving robotic arm grasping and mechanical reasoning, local high-fidelity representations such as point clouds, Mesh, or Surfel need to be bound within object-centric 3D bounding boxes to provide smooth normals and continuous gradients. For high-level natural language instruction understanding and long-term memory requirements, open-vocabulary scene graphs (such as ConceptGraphs or 3D DSG) fused with visual foundation models can be deployed at the top level, with node iteration and forgetting mechanisms to cope with the dynamic evolution of real environments. When systems rely on visual servoing or need to use simulation data for closed-loop policy training, 3DGS and NeRF systems that feature both explicit geometric tracking and photorealistic rendering capabilities are cutting-edge solutions that balance high fidelity and differentiable optimization.

As shown in Table 4 various typical map representations present clear technical complementarity in data structures, advantages and limitations, and application scenarios, providing comprehensive theoretical support and engineering reference for map selection and scheme design in embodied intelligence hardware-software systems.

Whereas Table 4 compares the qualitative strengths, limitations, construction methods, and application scenarios of each representation, Table 5 provides a quantitative, task-oriented view of memory overhead, update latency, geometric accuracy, computational cost, and adaptability to manipulation and language-guided navigation tasks, which is largely absent from prior surveys.

Occupancy grids occupy a few MB at 0.05 m resolution; 3DGS scenes typically contain 10^5^–10^6^ Gaussians (hundreds of MB); NICE-SLAM uses multi-scale feature grids of a few hundred MB with a high compression ratio; neural implicit online optimization is seconds per frame, while 3DGS renders at 30–100 fps.

## 3. Map Construction Methods

Map construction is the core component of robot environment modeling, aiming to continuously fuse multi-source sensor observations into a unified map representation while accurately estimating the robot’s own motion state. From a technical process perspective, map construction can be broadly summarized as two main threads: State Estimation and Map Updating. The former estimates robot trajectories through odometry, front-end registration, loop closure detection, and back-end graph optimization; the latter incrementally integrates observation data such as LiDAR point clouds, depth images, or visual features into the map based on optimized poses, and completes map consistency maintenance. In multi-sensor systems, it is also necessary to address key issues such as sensor spatiotemporal synchronization, extrinsic calibration, noise modeling, and multi-source information fusion to ensure the accuracy and robustness of mapping results.

As map representation forms continue to evolve, the core issues focused on during the construction process for different types of maps have gradually shown differentiated characteristics. 2D grid maps mainly focus on probabilistic occupancy modeling and efficient path planning; 3D voxel maps focus on volumetric fusion, sparse storage, and distance field construction; feature maps and mesh maps place greater emphasis on geometric expression, registration accuracy, and surface reconstruction; topological maps and semantic maps further introduce environmental structure abstraction, object-level semantics, and scene relationship modeling. In recent years, neural implicit maps, 3DGS maps, and hybrid maps have introduced neural representation, differentiable rendering, multimodal semantic fusion, and long-term map maintenance into the mapping process, gradually evolving map construction from traditional geometric reconstruction to comprehensive environmental modeling integrating geometry, semantics, and cognitive information.

**Figure 3:**
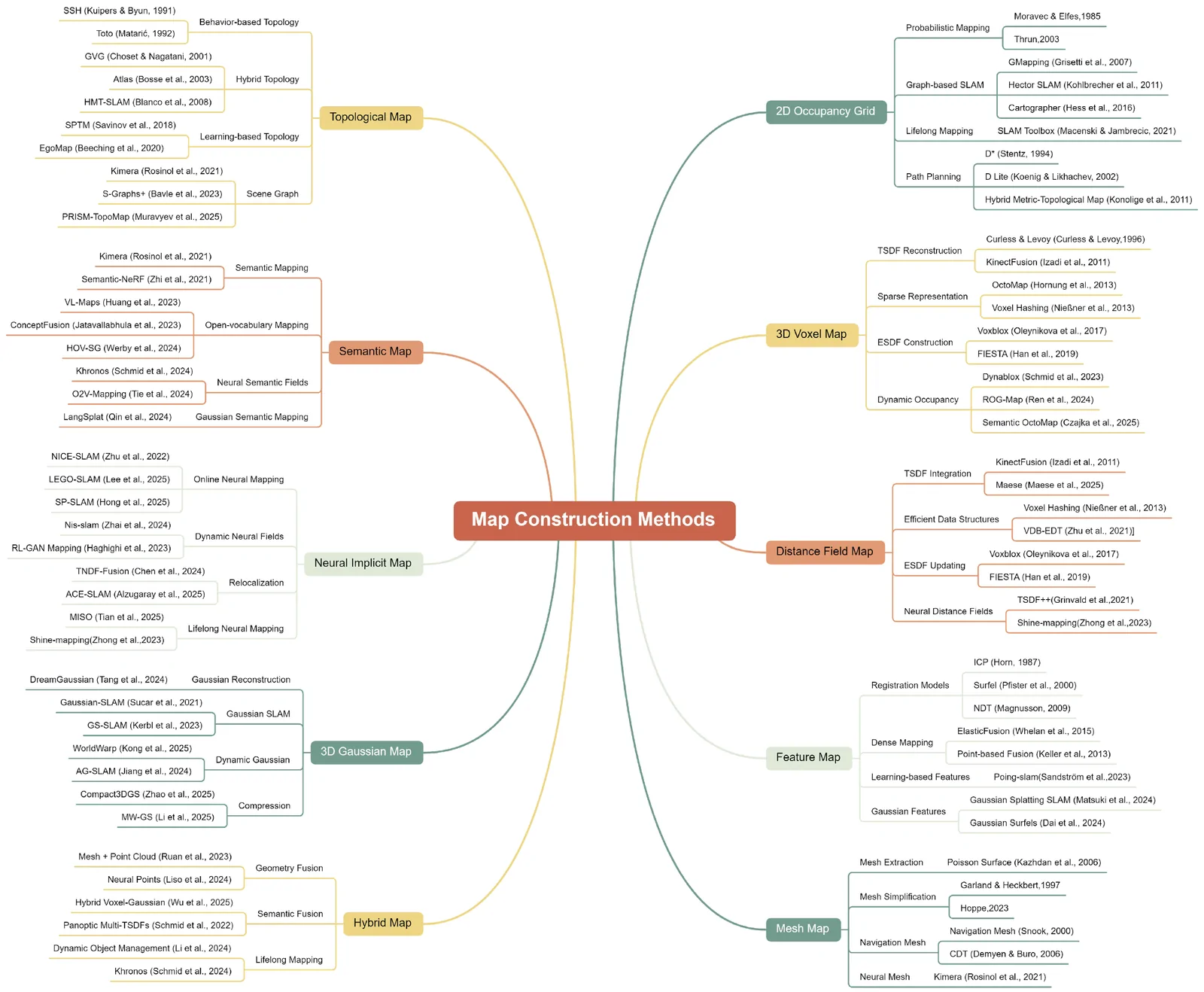
Typical algorithms for various map representations. References for the works shown, grouped by category: 2D occupancy grids [10,12,13,14,41,83,84,85,87]; Voxel maps [16,17,20,21,22,89,90,91,92]; Distance fields [17,21,89,90,93,94,95,96]; Feature maps [26,27,56,57,74,97,98,99]; Mesh maps [32,33,100,101,102,103]; Topological maps [32,36,37,38,40,82,104,105,106,107]; Semantic maps [15,32,108,109,110,111,112,113]; Neural implicit maps [44,96,114,115,116,117,118,119,120]; 3D Gaussian maps [45,48,121,122,123,124,125]; Hybrid maps [34,52,53,112,126,127].

Therefore, this Section first introduces the general SLAM process of map construction and its key component modules. Based on this, in conjunction with the ten typical map representations proposed in Section 2, it respectively reviews the typical construction methods, key technology evolution, and representative works for 2D grid maps, 3D voxel maps, distance field maps, feature maps, mesh maps, topological maps, semantic maps, neural implicit maps, 3DGS maps, and hybrid maps. Figure 3 shows representative algorithms for each map type, laying the foundation for the subsequent analysis of map updating mechanisms and long-term map management.

### 3.1. 2D Grid Map Construction

2D grid maps, as the starting point of robot spatial cognition, evolved around probabilistic fusion, global optimization, and path search efficiency improvement. In terms of probabilistic mapping and fundamental models, Moravec and Elfes pioneered the probabilistic fusion model for 2D sonar occupancy grid maps [10], and Thrun subsequently introduced forward generative models and EM algorithms, thoroughly resolving spatial dependency conflicts caused by the traditional grid independence assumption [41]. In the global optimization phase, GMapping introduced adaptive proposal distributions to reduce particle depletion [12], while Hector utilized multi-resolution grids to achieve high-frame-rate matching without odometry dependence [83]. With Cartographer adopting branch-and-bound optimization for large-scale real-time loop closure [13], 2D mapping gradually matured. Notably, SLAM Toolbox pioneered the ‘lifelong mapping’ concept, supporting long-term map dynamic merging and reloading for single robots through pose graph serialization [14]. Regarding advanced path planning, Stentz proposed D*, which avoids repeated global planning through local cost propagation [84]. Koenig and Likhachev subsequently introduced D* Lite, enabling efficient incremental replanning with a simpler implementation and theoretical guarantees [85]. Harabor and Grastien further proposed JPS, which accelerates A* search through forced-neighbor detection and symmetry reduction [86].Together, these methods improved the efficiency of grid-based navigation. Ultimately, Konolige et al.’s hybrid metric-topological structure [87] and Lau et al.’s wildfire method for local ultra-fast updates [88] effectively broke through the memory and scalability bottlenecks of traditional large-scale 2D mapping.

Overall, 2D grid maps have evolved from early static occupancy representations to foundational map frameworks supporting long-term operation. Future development will focus not merely on mapping accuracy, but on dynamic environment updating, lifelong map maintenance, multi-layer costmap construction, and path planning acceleration, continuing to serve as the foundational support for single-robot localization, navigation, and global planning in coordination with 3D maps and semantic maps.

### 3.2. 3D Voxel Map Construction

As early geometric foundations, Meagher and Connolly established octree encoding and incremental accumulation [18,128]. Subsequently, Chien et al. proposed an efficient tree conversion method for directly generating voxel and surface octrees from range data [129]. Curless and Levoy pioneered the Truncated Signed Distance Field (TSDF), achieving ultra-fast volumetric fusion of complex 3D depth images [22]. Levoy et al. successfully extended TSDF voxel grids to high-precision 3D scanning fusion of ultra-large-scale cultural heritage [130]. Lefebvre and Hoppe introduced perfect spatial hashing, providing boundless storage inspiration for subsequent extremely sparse 3D voxel maps [131]. Izadi et al. first utilized GPU and TSDF to achieve real-time dense 3D reconstruction [21]. To break rigid memory limits, Hornung et al. established probabilistic 3D occupancy standards [16], and Niessner et al. introduced spatial hashing technology, completely liberating the storage constraints of large-scale real-time 3D fusion [89]. Zollhofer et al. proposed performing shadow-based inverse rendering directly on TSDF voxel fields, recovering sub-millimeter 3D surface geometric details [132]. For computationally constrained UAV platforms, Oleynikova et al. achieved ultra-fast incremental ESDF updates on pure CPU [17]. Katyal et al. introduced generative fully convolutional networks, achieving prediction and structural completion of unobserved 3D occupancy voxel regions for the first time [133]. Han et al. proposed FIESTA, utilizing breadth-first search with an independent dual-queue mechanism for extremely fast incremental ESDF voxel map updates in dynamic environments [90]. Duberg and Jensfelt achieved significant memory compression and primitive insertion speed improvement by explicitly expressing unknown space [19].

In recent years, the research focus has shifted toward dynamic environment adaptation and underlying computational breakthroughs. Zhu et al. proposed VDB-EDT, an efficient Euclidean distance transform algorithm based on the sparse VDB data structure [93]. Cai et al. compressed dynamic 3D spatial update time to 4 percent of static trees [134]. Ren et al. proposed ROG-Map, constructing a robot-centric sliding window local voxel map that greatly reduces the memory explosion risk in large-scene flight [20]. Schmid et al. proposed Dynablox, achieving real-time detection and filtering of various dynamic objects in complex voxel environments based on TSDF and free space tracking [91]. Xi et al. proposed R-VoxelMap, introducing geometry-driven recursive plane fitting strategies within voxels to significantly improve the anti-noise accuracy of online 3D voxel mapping [135]. Li et al. proposed SliceOcc, which represents indoor 3D space using vertical slices for vision-based semantic occupancy prediction [136]. Huang et al. proposed Gaussian-Former, which represents scenes using 3D Gaussians for vision-based semantic occupancy prediction [137]. Chen et al. designed a dedicated caching mechanism from the underlying hardware architecture, breaking through the memory access performance bottleneck of 3D occupancy mapping in one stroke [138]. Czajka et al. compared different image segmentation methods for generating high-resolution Semantic OctoMaps [92].

3D occupancy maps are continuously evolving toward sparsification, high resolution, and dynamic adaptation. In the future, they will further integrate efficient storage structures such as spatial hashing, octrees, and hardware-software co-optimization to achieve real-time mapping and updating in larger-scale environments. At the same time, combining semantic perception, dynamic target removal, and open-world scene understanding will enable occupancy maps not only to describe spatial geometry but also to support long-term autonomous navigation and environmental perception tasks.

### 3.3. Distance Field Map Construction

The evolution of distance fields began with improvements in fusion efficiency. After Newcombe pioneered GPU real-time dense fusion [21], Maese et al. achieved constant-time CPU point cloud fusion through bitmask operations [94]. To address the memory disaster, data structures evolved from Niessner et al.’s hash-based voxel allocation [89] and Klingensmith et al.’s spatially hashed signed-distance fields [61] to Vizzo et al.’s flexible VDB-based TSDF integration [139] and Vespa et al.’s efficient octree-based volumetric mapping [140]. To directly serve robot obstacle avoidance, the direct conversion from TSDF to ESDF became a necessity: Oleynikova et al. created local wavefront diffusion [17], Han et al. compressed dynamic expansion nodes [90], Pan et al. eliminated ray-casting distance distortion [23], and Millane et al. reconstructed GPU concurrent pipelines [24]. In handling loop closure and dynamic deformation, where traditional pose graphs fail, non-rigid alignment techniques emerged: Whelan et al. [141], Dai et al. [142], and Newcombe et al. [143] respectively proposed elastic stretching surfels, historical weight revocation, and continuous 3D deformation field estimation; Slavcheva et al. further introduced differential geometry vector fields to handle drastic topological changes [144,145]. Currently, Grinvald et al. [95], Zhong et al. [96], and Yang et al. [146] have further advanced distance fields toward multi-object semantic encoding and implicit neural mapping stages, overcoming the geometric aliasing and catastrophic forgetting of traditional grids.

Distance field maps are gradually evolving from geometric reconstruction tools to unified environmental models integrating mapping, localization, obstacle avoidance, and motion planning. The future development trend is to further improve dynamic update efficiency, integrate semantic information and neural implicit representations, and leverage GPU and dedicated hardware to achieve real-time optimization, providing more precise and efficient continuous spatial representations for single robots.

### 3.4. Feature Map Construction

The evolution of feature maps centered on finding more efficient spatial primitives that better characterize physical surfaces. After the registration mathematical foundation was established by Horn [26], Pfister et al. [97] abandoned polygons and pioneered Surfel primitives. Biber and Straßer [25], Magnusson [27], Rusinkiewicz and Levoy [147], and Pauly et al. [148] successively proposed point-to-plane ICP and NDT probability density models, establishing matching robustness. Entering the real-time dense reconstruction era, Zhang and Singh [28] and Levinson and Thrun [149] set low-drift localization benchmarks; since rigid voxels are difficult to deform, Whelan et al. [56], Keller et al. [57], and Yan et al. [150] demonstrated the tremendous advantages of Surfels in elastic loop closure and uncertainty encoding; Behley and Stachniss [29], Schöps et al. [151], and Wang et al. [152] further utilized spherical projection, asynchronous mesh extraction, and superpixel dimensionality reduction to achieve large-scale system lightweighting. With the intervention of deep learning, physical distortion was decoupled: Ye et al. used learned descriptors with 3D Surfel maps for visual relocalization [153], while Dellenbach et al. introduced elastic LiDAR odometry to address motion distortion and improve registration accuracy [154]. Sandström et al. further anchored learnable implicit features to neural point clouds for dense tracking and mapping [98]. After 2024, 3D Gaussian Splatting reshaped primitive expression: Keetha et al. [50] and Matsuki et al. [74] established the photometric optimization paradigm for Gaussian SLAM; Fan et al. [30] and Dai et al. [99] perfectly integrated differentiable rendering with the physical properties of Surfel precise alignment by forcibly compressing the Gaussian *Z*-axis; at the same time, Kim et al.’s [155] silicon-level custom accelerator, Dai et al.’s [156] cross-view feature fusion, and Wang et al.’s [157] hierarchical adaptive nodes thoroughly solved the robustness challenges of Gaussian maps under extreme noise and computational constraints.

The development of feature maps is continuously evolving from traditional handcrafted features to deep learning features and learnable spatial primitives. In the future, greater emphasis will be placed on joint optimization of geometric, photometric, and semantic features, combined with novel representations such as Surfels, neural point clouds, and 3D Gaussians, to achieve integrated localization, mapping, relocalization, and rendering. Meanwhile, lightweight representation and long-term stable maintenance will also become important research directions for single-robot continuous operation.

### 3.5. Mesh Map Construction

Mesh maps reduce grid redundancy through topological connectivity, and their development has gradually progressed from geometric extraction toward higher-level environmental representation. Fowler and Little [158] investigated irregular triangulated terrain models, while Arkin [159] explored geometric path planning. Subsequently, point-cloud processing and Marching Cubes techniques provided practical foundations for surface reconstruction [31,60]. To address the computational complexity of large-scale meshes, Hoppe [100], Garland and Heckbert [101], and Isenburg et al. [160] respectively developed progressive meshes, quadric error metrics, and processing-sequence methods for efficient mesh simplification. Meanwhile, Snook [102], Kallmann et al. [161], and Demyen and Buro [103] advanced navigation meshes, dynamic constrained Delaunay triangulation, and triangulation-based pathfinding. Kazhdan et al. developed Poisson-based methods for watertight surface reconstruction [33,162], while Teixeira and Chli [163], Cui et al. [164], and Piazza et al. [165] investigated real-time mesh estimation, navigation-mesh pathfinding, and CPU-based large-scale mesh reconstruction, respectively. Subsequently, Schöps et al. developed online Surfel-based mesh reconstruction [151], Grinvald et al. introduced multi-object tracking and reconstruction into TSDF maps [95], and Rosinol et al. presented Kimera as a real-time metric-semantic mapping framework that integrates geometric meshes with semantic information [68]. With the development of neural scene representations, Zhu et al. [44] and Sucar et al. [45] explored implicit dense mapping, while Ruan et al. [34], Zheng et al. [35], and Matsuki et al. [74] respectively investigated real-time LiDAR meshing, Gaussian-based SLAM, and Gaussian Splatting SLAM in dynamic environments.

The development focus of mesh maps has expanded from high-precision geometric reconstruction to dynamic updating, semantic fusion, and real-time planning. In the future, differentiable rendering, neural implicit representation, and Gaussian reconstruction technologies will be further combined to achieve online generation and continuous maintenance of high-fidelity meshes. At the same time, by introducing object-level semantics and scene graph structures, mesh maps will gradually serve as important carriers for robot environmental understanding and complex task reasoning.

### 3.6. Topological Map Construction

Topological maps abstract environments into connectivity relationships through node-edge structures, evolving from early liberation from geometric coordinate constraints to hierarchical cognitive maps integrating metric, semantic, and learning capabilities. Kuipers and Byun first proposed the Spatial Semantic Hierarchy (SSH) model, which directly extracts local extrema as topological nodes through low-level sensor feedback, laying the foundation for behavior-driven topological mapping [37]. Matarić bound topological nodes to robot behaviors, utilizing subsumption architecture and spreading activation for navigation planning, enabling topological maps to possess autonomous behavior expression capability for the first time [104]. Subsequently, Shatkay and Kaelbling introduced Hidden Markov Models (HMMs) into topological mapping, mitigating false loop closures caused by perceptual aliasing through temporal sequence statistical learning [166]; Thrun further combined EM algorithms to automatically partition topological regions on metric maps, achieving the unification of probabilistic mapping and topological abstraction [167].

As environmental scale continuously expanded, research focus gradually shifted toward collaborative modeling of topological and metric maps to balance global planning capability and local localization accuracy. Fabrizi and Saffiotti utilized mathematical morphology and digital topology to directly extract open spaces and corridor structures from grid maps, achieving topology generation without manual feature design [168]. Choset and Nagatani proposed pure topological SLAM based on Generalized Voronoi Graphs (GVGs), effectively avoiding accumulated drift through free-space skeleton navigation [36]. Tomatis et al. combined POMDP with EKF, delaying incorrect loop closure decisions through multi-hypothesis reasoning [169]; Bosse et al. proposed the Atlas framework combining local submaps with topological connections, breaking through the bottleneck of global covariance growth [105]; Blanco et al. further achieved joint optimization of local metric maps and global topological structures within a unified Bayesian framework, making hybrid maps an important paradigm for long-distance SLAM [38].

After completing metric fusion, topological maps began to evolve toward high-level semantics and task reasoning. Galindo et al. first constructed a three-layer map structure integrating metric, topological, and semantic concept layers, enabling robots to perform advanced logical reasoning and task planning [170]. Konolige et al. proposed a hybrid navigation framework where metric maps are only generated locally while global planning completely relies on topological graphs, significantly reducing large-scale environment mapping costs [87]. Tully et al. maintained multiple topological connection structures through hypothesis forests, improving robustness during loop closure phases [171]; Blochliger et al. directly generated convex free-space clusters from sparse visual point clouds, achieving topological navigation without dense reconstruction [39].

In recent years, deep learning has further driven topological maps from manual construction toward autonomous learning. Savinov et al. proposed semi-parametric topological memory networks, replacing explicit geometric modeling with image reachability learning to achieve end-to-end topological navigation [106]. Beeching et al. projected CNN visual features onto local topological-metric maps, introducing spatial geometric constraints to neural networks [107]. Chaplot et al. introduced explicit topological graphs into reinforcement learning frameworks, enhancing unknown region exploration capability through virtual nodes [172].

As robot cognitive demands continue to increase, topological maps have further evolved into high-level semantic representations such as Scene Graphs. Rosinol et al. constructed 3D Dynamic Scene Graphs (DSGs) integrating metric meshes, dynamic objects, and room-level relationships, enabling topological maps to possess object-level cognitive capability [32]. Bavle et al. organized keyframes, walls, rooms, and floors into a four-layer topological factor graph, constraining low-level SLAM optimization with architectural hierarchies [82]. Muravyev et al. proposed a lightweight coordinate-free topological mapping framework, completing rapid 3D point cloud association through 2D projection to achieve online topological maintenance [40]. Abu-Jassar et al. combined a neural-network component with stochastic optimization to construct adaptive trajectories for mobile robots, illustrating the application of learning-based optimization to robot path generation and navigation decision-making [173].

Topological maps are gradually evolving from traditional node-edge navigation structures to hierarchical cognitive representations integrating geometry, semantics, and task information. Future development will place greater emphasis on deep integration of topological and metric maps, combined with scene graphs, reinforcement learning, and large model reasoning capabilities to achieve autonomous generation, online maintenance, and dynamic optimization of topological structures, providing higher-level environmental abstraction for single-robot long-term navigation, exploration, and task planning.

### 3.7. Semantic Map Construction

Semantic maps have evolved from fixed-category annotation to open-vocabulary, multimodal fusion, and continuous semantic field representation. Early methods grounded topological maps in metric-semantic fusion: Rosinol et al. first extended topological maps into 3D Dynamic Scene Graphs (DSGs) integrating metric meshes, dynamic humans/objects, and room-level relationships, enabling topological maps to evolve from spatial connectivity structures to cognitive maps supporting high-level semantic reasoning [32]. Subsequently, open-vocabulary semantics began to be integrated into map representations. Huang et al. densely embedded CLIP features into 2D maps, enabling robots to perform zero-shot object retrieval and navigation based on natural language descriptions [15]; Jatavallabhula et al. fused visual, textual, and geometric information, achieving multimodal concept queries of 3D maps without additional training [108]. Werby et al. further established hierarchical topological relationships among objects, rooms, and floors in scene graphs, combined with Voronoi diagrams to support cross-floor language navigation [109]; Wang et al. utilized adaptive octrees to construct object-level open-vocabulary scene graphs, enhancing large-scale spatial reasoning capability while reducing storage overhead [42].

With the development of neural implicit representations, research began to introduce continuous semantic fields into map construction. Zhi et al. first added semantic rendering heads to NeRF, achieving continuous dense 3D semantic reconstruction [110]. Tie et al. fused language features with voxel geometry into open-vocabulary neural implicit fields, utilizing adaptive voxels to achieve dynamic online 3D semantic mapping [111]; Schmid et al. further unified short-term dynamic changes and long-term scene evolution, jointly optimizing spatiotemporal semantic maps within the same factor graph framework [112].

In recent years, 3D Gaussian Splatting (3DGS) has further advanced semantic mapping through the integration of language features into Gaussian representations. Qin et al. introduced LangSplat, which combines 3D Gaussian representations with compact language features to enable efficient open-vocabulary querying [113]. Taken together, the methods reviewed in this section illustrate three complementary directions in semantic map construction: open-vocabulary language grounding, continuous neural semantic fields, and efficient Gaussian-based semantic representations. Semantic maps are progressively developing from fixed-category annotation toward open-vocabulary, multimodal fusion, and continuous semantic field representation. In the future, with the support of vision-language foundation models, natural language-driven open-world semantic understanding will be achieved, further integrating 3D scene graphs, neural implicit representations, and 3D Gaussian maps to enable robots to possess long-term environmental cognition, semantic reasoning, and human–robot interaction capabilities, driving maps from geometric representations toward cognitive representations.

### 3.8. Neural Implicit Map Construction

Recent studies on neural and learnable map representations have addressed se-mantic integration, loop closure, dynamic-scene modeling, memory efficiency, re-al-time reconstruction, and uncertainty estimation. SNI-SLAM incorporated semantic information into neural implicit SLAM [174], while SLC2-SLAM employed shared la-tent codes for semantic-guided loop closure [175]. HI-SLAM investigated hierarchical implicit representations [176]. DNS-SLAM integrated semantic information into dense neural mapping [177]. OpenGS-SLAM subsequently extended learnable map representations to open-set, object-level scene understanding using 3D Gaussian Splatting [178], while NIS-SLAM combined neural implicit mapping with semantic information for consistent 3D scene understanding [114]. For dynamic-scene representation, Zhong et al. used a 4D implicit neural representation for LiDAR mapping in dynamic environments [179], while Weng et al. employed neural implicit representations to con-struct digital twins of unknown articulated objects [180]. Haghighi et al. investigated neural implicit dense semantic SLAM [115], while Zhou et al. proposed a memory-efficient implicit scene representation for endoscopic RGB SLAM [181]. In parallel with advances in neural implicit mapping, Davies et al. combined two-dimensional occupancy grid mapping with deep-learning-based refinement to improve conventional SLAM map representations [182]. Recent studies have improved neural mapping through scene priors [116], feed-forward monocular reconstruc-tion [46], 3D reconstruction priors [183], and scene-coordinate regression [117]. iNeRF estimates camera poses from pretrained radiance fields [184], while BARF jointly optimizes poses and neural fields [185]. Subsequent methods address loop closure [126], point-based globally consistent LiDAR mapping [51], multiresolution submap optimization [118], and ORB-assisted NeRF mapping [186]. NeRF-SLAM enables real-time dense monocular mapping [47], while TNDF-Fusion supports large-scale LiDAR mapping and localization [119]. Other advances include quantized neural SLAM [187], multiscale neural feature fields [188], uncertainty-aware NeRF [189], and NeRF-based dense visual SLAM [190].

The development of neural implicit maps is gradually progressing from high-precision reconstruction toward real-time mapping, continual learning, and lifelong maintenance. Future research will focus on addressing key issues such as low online optimization efficiency, catastrophic forgetting, and dynamic environment adaptation, integrating large model priors, incremental learning, and lightweight network structures to achieve high-fidelity, high-efficiency, low-resource-consumption continuous implicit environmental representations, providing a unified neural map framework for single-robot long-term autonomous operation.

### 3.9. 3D Gaussian Splatting Map Construction

3DGS enables efficient scene representation through differentiable rasterization. Li et al. explored pose-free generalizable reconstruction [191], while Feng et al. investigated diffusion-based single-view reconstruction [192]. Guo et al. developed open-vocabulary scene understanding [193], Lee et al. integrated language features into Gaussian SLAM [120], and Zeller and Wu investigated camera localization [194]. Tang et al. introduced DreamGaussian for efficient 3D content generation [121]. Huang et al. addressed reconstruction from long image sequences [195], Feng et al. accelerated large-scale rendering [196], and Ren et al. improved training efficiency [197]. Chang et al. investigated sparse-view surface reconstruction [198], while Ma et al. combined meshes and Gaussians for dynamic object reconstruction and simulation [199]. Gong et al. reviewed 4D scene reconstruction [200], and Pan et al. proposed controllable 4D scene generation [201].

Further advances include Lee et al.’s Gaussian compression [202], Hong et al.’s multi-sensor mapping [203], Guo and Wang’s SDF-based surface reconstruction [204], Park et al.’s combined 2D–3D Gaussian reconstruction [205], and Li et al.’s Chorus framework for multi-teacher pretraining of a holistic 3D Gaussian scene encoder [206], Kong et al. investigated geometry propagation [123], Zhao et al. developed compact Gaussian SLAM [124], and Jiang et al. studied active Gaussian SLAM [125]. Rosinol et al.’s hierarchical scene graphs provide a potential framework for connecting Gaussian maps with semantic reasoning [32], while Na et al. investigated transparency through Gaussian radiance transport [207].

**Figure 4:**
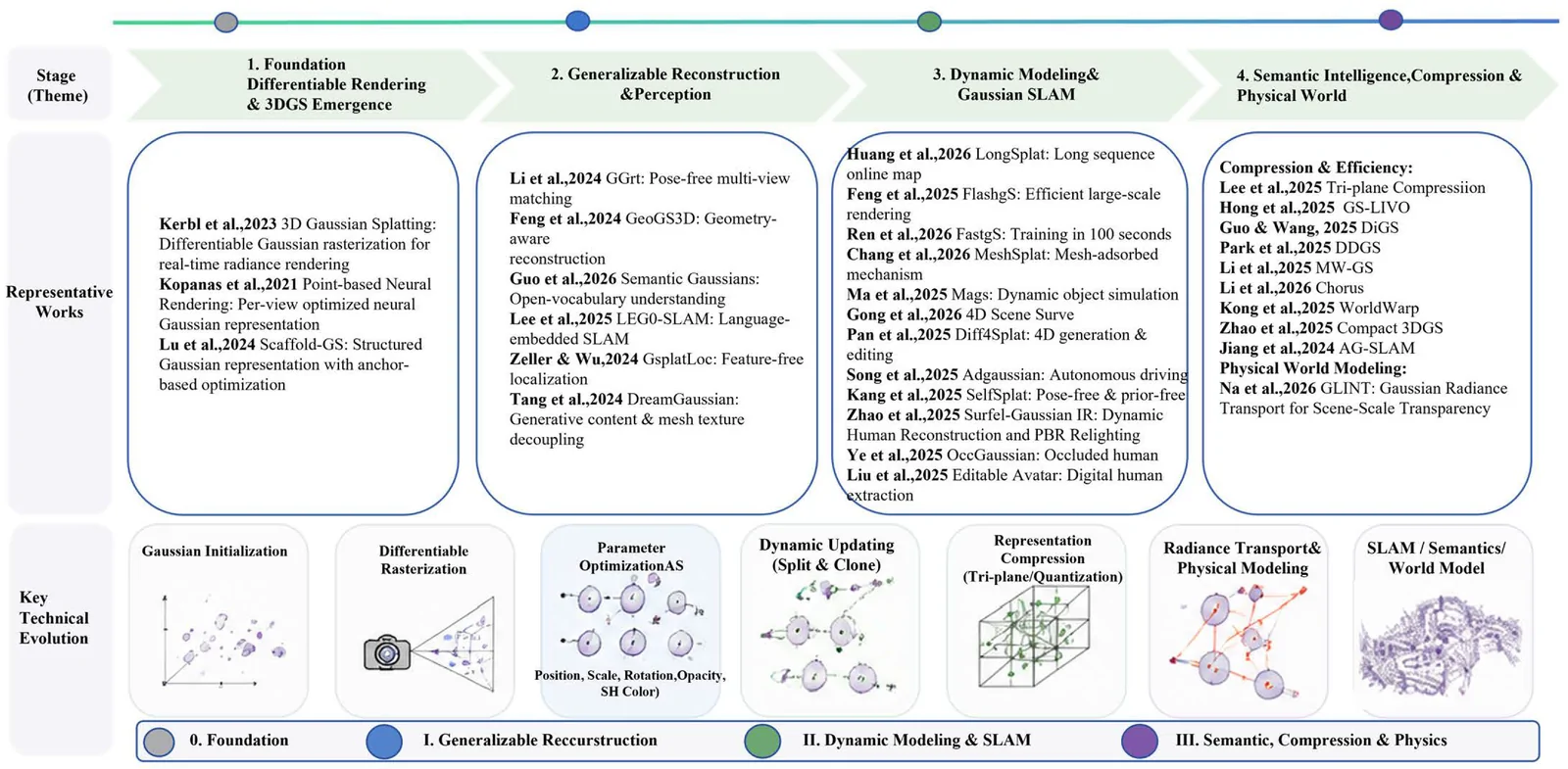
The development of 3D Gaussian Splatting maps. References for the works shown, grouped by category: Foundation [48,79,208]; Generalizable reconstruction and perception [120,121,191,192,193,194]; Dynamic modeling and Gaussian SLAM [195,196,197,198,199,200,201,209,210,211,212,213]; Compression and physical world modeling [122,123,124,125,202,203,204,205,206,207].

3D Gaussian Splatting maps are rapidly expanding from high-quality novel-view rendering to real-time SLAM, semantic mapping, and dynamic scene modeling. Figure 4 shows their development trend. Future development will focus on Gaussian primitive compression, real-time incremental updating, multimodal semantic fusion, and physically consistent modeling, further combining foundation models to enhance open-world generalization capability, gradually becoming a new-generation map representation form that integrates mapping, localization, rendering, and cognitive functions.

### 3.10. Hybrid Map Construction

Single data structures can no longer meet the complex requirements of modern autonomous systems. Hybrid maps fuse various heterogeneous geometric maps such as triangular meshes and point clouds, balancing fine surface extraction and real-time localization. The core design principle of current architectures is to strictly ensure efficient map updating and consistency maintenance for a single robot throughout its entire lifecycle. For real-time geometric mapping, Ruan et al. proposed SLAMesh, which jointly per-forms LiDAR localization and incremental mesh reconstruction to generate continuous surface maps from point-cloud observations [34]. To improve geometric representation and mapping efficiency, Pan et al. [51] and Sandström et al. [98] explored point-based neural representations for localization and dense mapping. Keetha et al. proposed SplaTAM, which uses 3D Gaussian primitives for dense RGB-D tracking and mapping [50]. Wu et al. developed a semantic-driven voxel representation that combines Gaussian and planar features for LiDAR–inertial odometry [52]. Long-term evolution management for single robots (e.g., short-term motion decomposition and object-level isolation [53,112,127]) is discussed in Section 4.

In the autonomous driving domain, hybrid map construction ideas and application forms are more extensive, combining Vector Maps, 3D Occupancy Grids, HD Point Clouds, and Topological Lane Graphs to construct hierarchical multimodal spatial representations. The fusion of vector lane topology and dense metric geometry couples vector maps describing traffic rules and lane topology relationships (HD Vector Map) with underlying 3D point cloud/voxel maps, providing both high-level topological constraints for vehicle behavior planning and centimeter-level precise physical geometric constraints. For example, HoMap and MapTRv2 employ end-to-end frameworks to construct online vectorized HD maps and model the spatial relationships among road elements [214,215]. Semantic occupancy grids and Gaussian representations provide complementary in-formation about road structures, obstacles, and dynamic objects. Wang et al. introduced OpenOccupancy, a large-scale benchmark that provides dense 3D annotations and standardized evaluation protocols for surrounding semantic occupancy perception [216]. Zhou et al. proposed DrivingGaussian, which employs composite Gaussian splatting to reconstruct dynamic surrounding driving scenes and achieve high-quality novel-view rendering [217]. These studies represent two complementary directions in autonomous-driving environment representation: dense semantic occupancy perception and photorealistic dynamic-scene reconstruction. For large-scale autonomous-driving scenes, online mapping systems commonly separate local geometric perception from global vectorized map representation. HDMapNet constructs vectorized HD maps from onboard sensor observations [218], while StreamMapNet introduces temporal propagation to improve the consistency and efficiency of streaming online HD-map construction [219].

Hybrid maps represent the future development direction of single-robot map representations, with the core goal of integrating the advantages of various representations including grids, voxels, distance fields, meshes, topology, semantics, neural implicit, and 3D Gaussian within a unified framework, achieving efficient collaboration between different map layers. In the future, greater emphasis will be placed on cross-representation dynamic updating, long-term consistency maintenance, unified map management, and task-oriented on-demand expression, ultimately constructing a lifelong map system that can simultaneously support localization, navigation, perception, interaction, and intelligent decision-making.

### 3.11. Concluding Remarks on Map Construction

This Section systematically reviewed the construction methods of current mainstream map representations and their development trajectories, centered on single-robot environmental map construction. From early 2D grid maps, 3D voxel maps, and distance field maps, to feature maps, mesh maps, topological maps, semantic maps, and to recently emerging neural implicit maps, 3DGS maps, and hybrid maps, various map representations have continuously broken through the limitations of traditional geometric modeling, gradually achieving evolution from 2D to 3D, from discrete to continuous, from geometric to semantic, from static to dynamic, and from single representation to multi-representation fusion.

Overall, different map representations have formed their respective advantages for different application needs. Grid maps, voxel maps, and distance field maps possess characteristics of maturity, stability, and high query efficiency, serving as important foundations for localization, obstacle avoidance, and path planning; feature maps and mesh maps have relatively high advantages in geometric reconstruction accuracy and real-time mapping; topological maps and semantic maps further enhance environmental abstraction capability, gradually enabling maps to possess task reasoning, human–robot interaction, and high-level decision support capabilities; neural implicit maps and 3DGS maps break through traditional discrete storage methods, demonstrating tremendous potential in continuous scene expression, high-fidelity reconstruction, and open-world mapping; hybrid maps, by fusing multiple map representations, achieve complementary advantages between different map layers, gradually becoming an important development direction for long-term autonomous robot map construction.

Meanwhile, as robotic applications gradually progress from static scenes to long-term operation and open dynamic environments, map construction has not only focused on generating environmental geometric information but increasingly emphasizes the continuous maintenance capability of maps throughout their lifecycle. How to achieve real-time updating in dynamic environments, long-term consistency maintenance, cross-representation collaborative management, and low resource consumption while ensuring mapping accuracy has become an important topic in current map research. Therefore, the development focus of map construction technology is gradually shifting from ‘how to construct maps’ to ‘how to continuously maintain and efficiently update maps.’ The next Section will discuss map updating mechanisms, systematically analyzing the update strategies and key technologies of different map representations, and further summarizing the development trends and future directions of single-robot map updating.

## 4. Map Update and Management for Single Robots

In the tasks of long-term autonomous operation and Lifelong SLAM for single robots, the dynamic changes in the environment, sensor observation noise, hardware storage limitations on map size, and inevitable accumulated pose errors collectively constitute the core challenges of underlying map maintenance. Traditional SLAM systems are typically based on the ‘static world assumption’; however, in the real physical world, environments evolve continuously over time. Without introducing efficient map update and management mechanisms, robots will encounter severe ‘map degradation,’ leading to localization failure and memory exhaustion.

**Figure 5:**
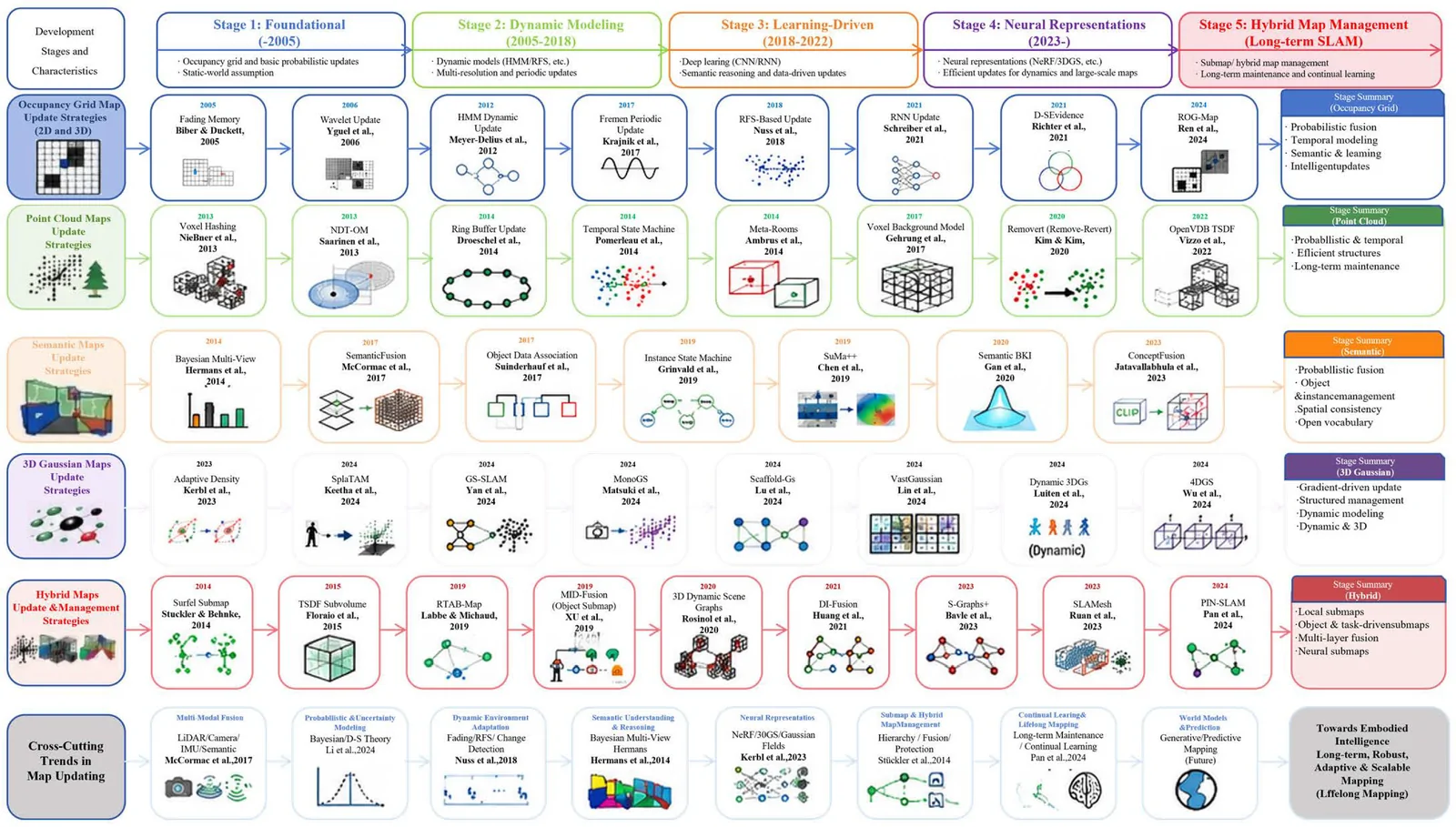
Typical algorithms for various map updating and management. References for the works shown, grouped by category: Occupancy grid updates [20,220,221,222,223,224]; Semantic surfel filtering [225]; Learning-based and evidential occupancy grid updates [226,227]; Point cloud updates [89,139,228,229,230,231,232,233]; Semantic updates [108,225,234,235,236,237,238]; 3D Gaussian up-dates [48,49,50,73,74,208,239,240]; Hybrid map management [34,51,82,241,242,243,244,245,246]; Cross-cutting trends [48,51,53,224,234,235,241].

With the rise of embodied intelligence, robots no longer merely need to solve low-level navigation problems of ‘where am I’ and ‘how to avoid obstacles,’ but also need to understand the environment through multi-sensor fusion perception, perform complex logical reasoning, and execute long-horizon physical interaction tasks (such as object rearrangement, semantic target search, etc.). Figure 5 shows the development trend of single-robot map updating and management. This trend toward task-driven evolution requires that the underlying maps must possess multimodal data fusion capability, lifecycle management capability across different timestamps, and long-term scheduling capability that breaks through hardware memory bottlenecks.

### 4.1. Updates and Management of Conventional Maps

In the perception system of embodied intelligence, ordinary maps of different dimensions serve distinct functions ranging from low-level safe obstacle avoidance to high-level semantic understanding. When operating in dynamic environments, a single robot must adopt customized updating and filtering strategies tailored to different data structures.

Most existing studies focus on specific map representations, single mapping frameworks, or dedicated application tasks. For instance, substantial progress has been made in the fields of 2D occupancy grids, 3D voxel maps, semantic maps, and neural implicit maps respectively [10,11,16,17,21,32,44,45,68]. In recent years, as robotic systems evolve toward large-scale and multi-robot collaboration, some studies have further investigated multi-agent map fusion, distributed SLAM, and collaborative environmental modeling. Nevertheless, these methods predominantly concentrate on mapping efficiency and consistency maintenance, while map lifecycle management and continuous knowledge accumulation during long-term operation remain underexplored [63,69]. In particular, the independent map lifecycle management of single-agent systems in long-term operating environments still requires further systematic investigation. For embodied intelligence, a single robot must be able to cope with environmental changes and incrementally update its local maps using onboard computational resources, particularly in challenging environments [247].

In recent years, several key works on long-term map management for single agents have emerged. For example, the experience-based navigation framework proposed by Churchill and Newman enabled robots to maintain and reuse multiple mapping experiences under changing environmental conditions [248]. SeqSLAM, developed by Milford and Wyeth [249], further demonstrated that a single robot can achieve robust long-term visual localization through sequence matching despite substantial seasonal and illumination changes. The Fusion++ system by McCormac et al. [250] illustrates how a single robot can construct object-based volumetric maps by incorporating semantic instances and independently manage the spatial lifecycle of these objects within a single-machine system. In neural implicit mapping, Kong et al. [251] proposed vMAP, an object-level RGB-D SLAM system that represents individual objects using separate compact neural fields and updates these models online. Here, “vectorised” refers to the parallel optimisation of multiple object-level neural field models, rather than the construction of conventional vector maps.

Against the backdrop of rapid advances in NeRF, 3DGS, open-vocabulary semantic mapping [43], and foundation models [252], new generations of map representations continue to emerge. Map systems for single agents have gradually evolved from one-time static mapping to long-term maintenance, continuous learning, and task-driven updating. However, there remains a relative scarcity of review articles that provide a unified analysis and summary of this complete technical chain. Therefore, it is necessary to systematically sort out the evolution of map representations, mapping methods, and independent map management and updating mechanisms, starting from the fundamental demand of long-term autonomous operation of single agents. A unified analytical framework covering the entire map lifecycle should be established to offer a more comprehensive reference for future research.

#### 4.1.1. Grid Map Update Strategies

Biber proposed the Fading Memory updating scheme, which adopts a multi-timescale updating strategy for dynamic environments. The map is divided into multiple layers, and the weight of historical observations gradually decreases with time according to the decay coefficient. This design allows moving or disappearing obstacles to be naturally overwritten by new observations, realizing continuous updating of long-term maps [220]. Subsequently, Yguel et al. put forward the Wavelet Mask updating mode, which leverages wavelet transform to perform multi-resolution updating on continuous homogeneous grids in the frequency domain. Breaking through the traditional per-grid updating paradigm, this method significantly improves the mapping efficiency of large open areas [221].

With in-depth research on environmental dynamics, Meyer-Delius et al. proposed a Hidden Markov Model (HMM) state transition updating scheme, which breaks the static environment assumption in traditional Bayesian mapping [222]. Each grid is modeled as a hidden Markov process with state transition probabilities, enabling grids to automatically shift from occupied to free states over time and better adapt to short-term dynamic environments. For long-running robots, Krajník et al. further proposed the Fourier-based periodic (FreMEn) updating scheme, which introduced frequency-domain analysis into map updating for the first time [223]. By applying Fourier transform to grid occupancy states to extract periodic patterns of environmental changes (such as diurnal cycles and peak pedestrian flow), the map can not only reflect the current state but also predict future environmental change trends.

To further enhance dynamic target processing capability, Nuss et al. proposed a Random Finite Set-based dynamic occupancy grid mapping method that uses particle-based representations to estimate occupancy and motion states [224].

Semantic information has also been used to handle dynamic objects in other map representations. For example, Chen et al. proposed SuMa++, a semantic LiDAR SLAM system that uses semantic consistency to filter unstable surfels associated with moving objects [225]. This approach operates on a surfel map rather than an occupancy grid. For occupancy-grid representations, Schreiber et al. proposed a recurrent neural net-work that processes sequences of measurement grid maps to estimate cell-wise occupancy probabilities and velocities [226]. By combining convolutional and recurrent layers, the method exploits spatial and temporal information to distinguish static and dynamic regions.

In parallel, uncertainty modeling has provided another direction for improving occupancy grid mapping. Richter et al. proposed a semantic evidential grid mapping framework for monocular and stereo cameras, explicitly representing uncertainty in vision-based range estimation [227]. The framework incorporates semantic infor-mation into an evidential grid representation and facilitates fusion with range-sensor data. 

Overall, the grid-based methods reviewed above address complementary aspects of map updating in changing environments. Fading-memory and HMM-based ap-proaches account for temporal changes in occupancy, while FreMEn models recurring environmental patterns. Wavelet representations support compact multi-resolution mapping, and random finite set methods estimate both occupancy and motion states. Recurrent neural networks exploit spatial and temporal information for learned dy-namic occupancy estimation, while evidential approaches support explicit uncertainty modeling. Together, these methods improve the adaptability of occupancy maps through temporal modeling, motion estimation, efficient representation, and uncer-tainty handling.

#### 4.1.2. Point Cloud Map Update Strategies

Nießner et al. first proposed the ultra-fast Voxel Hashing update scheme [89]. By using hash tables to directly map 3D voxel coordinates, it broke the computational bottleneck caused by frequent updates of deep nodes in traditional octrees, and achieved submap insertion and fusion with approximate O(1) time complexity at any spatial position, laying the foundation for real-time incremental updating of large-scale dense point cloud maps.

Subsequently, Saarinen et al. proposed the NDT-OM update scheme [228]. To address the limitation of traditional NDT maps in handling dynamic obstacles, he integrated the ray-casting occupancy grid model into the continuous 3D Normal Distributions Transform (NDT), developing a joint update mechanism of “probabilistic occupancy + Gaussian distribution”. This enables point cloud maps to erase moving targets in real time while maintaining high memory efficiency.

To further adapt to local map updating during the continuous motion of robots, Droeschel et al. proposed the Ring Buffer rolling update scheme [229]. The map maintained a fixed-size sliding window centered on the robot. As the robot moves, historical point clouds beyond the range are automatically moved out of the buffer, while new observation data are continuously rolled in, achieving real-time mapping over an infinite range under limited memory constraints.

Meanwhile, Pomerleau et al. proposed the Temporal State Machine update scheme for long-term map maintenance [230]. Aiming at the map aging problem faced by long-running robots, they assigned temporal states and confidence levels to points in the map, and continuously adjusted their credibility in combination with voxel ray tracing. Points that remain unsupported by observations for a long time and are continuously penetrated by new rays will gradually decay and be eventually deleted, realizing the dynamic renewal of the map.

Instead of continuously modifying the base map, Ambrus et al. further proposed the Meta-Rooms differentiated update scheme [231]. The robot retained independent temporal maps for each inspection tour, extracts newly added or disappeared objects through change detection, and manages them as independent entities rather than directly overwriting the original map. This fully preserves the evolution history of the environment across different time scales.

With the growing demand for mapping in dynamic environments, Gehrung et al. proposed the voxel background model and spatio-temporal filtering update scheme [232]. They constructd a volumetric background model that evolves over time, only fusing stable point clouds verified by continuous observations into the static map, while transient dynamic targets are automatically filtered out. This improves map stability in complex dynamic environments such as urban roads.

To address the problem of false deletion of dynamic point clouds, Kim and Kim proposed the Removert (Remove–Revert) update scheme [233]. This method first adopts an aggressive strategy to remove all point clouds suspected of being dynamic (Remove), then re-validates the deleted regions using multi-resolution depth maps, and recovers static structures mistakenly deleted due to occlusion or viewpoint changes (Revert), significantly improving the purity and completeness of offline static maps.

In recent years, sparse volumetric data structures have further advanced the efficiency of point cloud map updating. Vizzo et al. proposed a TSDF incremental update scheme based on the OpenVDB tree, which introduces sparse volumetric data structures from the film industry into robotic mapping [139]. It achieves highly adaptive sparse TSDF storage and fast incremental fusion, greatly reducing memory footprint and improving map update efficiency while ensuring the accuracy of high-resolution maps.

Point cloud map updating has gone through four stages: probabilistic occupancy updating, temporal maintenance, efficient data structure optimization, and long-term dynamic maintenance. Early methods realized the updating and removal of dynamic points through occupancy probability, temporal decay and state machine mechanisms. Afterwards, efficient data structures such as Voxel Hashing, Ring Buffer and VDB trees have driven map updating toward hash-based access, sliding window management and sparse voxel storage. In recent years, methods including Meta-Rooms and Removert have further integrated change detection, background modeling and spatio-temporal consistency constraints to achieve continuous maintenance in dynamic environments.

#### 4.1.3. Semantic Map Update Strategies

Hermans et al. first proposed the Bayesian multi-view probabilistic update scheme [234]. To address the high noise and frequent label fluctuation in single-frame semantic segmentation, this method maintains a category probability histogram for each voxel and continuously fuses multi-view observations via recursive Bayesian inference. It is further combined with 3D Conditional Random Fields (3D-CRFs) for spatial consistency optimization, achieving stable multi-view semantic map fusion.

Building on this work, McCormac et al. proposed the SemanticFusion recursive probabilistic fusion scheme [235]. It back-projects 2D semantic probabilities predicted by convolutional neural networks onto Surfel maps in real time, and replaces conventional label overwriting with continuous Bayesian probability accumulation. In this way, sporadic misclassifications will not compromise long-term semantic consistency, enabling real-time online semantic map updating.

As map representations evolved from semantic categories to semantic instances, Sünderhauf et al. proposed the object-level data association update scheme [236]. The system manages detected targets as independent objects, and continuously updates object poses, categories and attribute information through persistent data association. For unmatched targets, new object nodes are created automatically, supporting the continuous evolution of object-level semantic maps.

Furthermore, Grinvald et al. proposed the instance state machine update scheme in Voxblox++, which determines instance matching results by combining geometric overlap relations and semantic consistency [237]. Instance merging (Merge) is performed when new observations satisfy the association criteria; otherwise, new instances are spawned (Spawn). This achieves adaptive updating of instance boundaries and object states in dynamic scenes.

Alongside object-level association and instance management, maintaining semantic map consistency requires handling moving objects. Chen et al. addressed this issue in SuMa++, a semantic LiDAR SLAM system based on surfel maps [225]. During map updating, semantic inconsistency between incoming observations and the existing map introduces a penalty into the recursive update of surfel stability. Surfels that remain unstable over successive observations are subsequently removed. The system also incorporates semantic compatibility into projective scan matching to improve pose estimation in dynamic environments. Spatial consistency is another important aspect of semantic map maintenance. Gan et al. proposed Semantic BKI, a Bayesian kernel inference approach for spatially continuous semantic mapping [238]. This method leverages continuous spatial kernel functions to propagate single sparse semantic observations to the neighborhood probability field, and performs incremental probability updates in continuous space. It effectively improves the spatial consistency and generalization capability of semantic maps.

In recent years, with the advancement of vision-language models, semantic map updating has gradually shifted toward open-vocabulary representations. Jatavallabhula et al. proposed the ConceptFusion high-dimensional feature pooling update scheme [108]. It adopts high-dimensional CLIP visual–semantic features to replace traditional discrete category labels, achieves cross-view data association via cosine similarity in the latent space, and continuously fuses multi-view features using Exponential Moving Average (EMA). This enables maps to progressively accumulate open-vocabulary semantic representations, and provides support for cross-modal understanding and open-world reasoning.

Semantic map updating has undergone four stages: probabilistic fusion, object-level updating, spatially continuous optimization, and open semantic representation. Early methods centered on recursive Bayesian updating, and improved semantic label stability through multi-frame observation accumulation. Afterwards, object-level data association and instance management were introduced to realize continuous maintenance of dynamic targets and environmental information. Semantic-consistency-based surfel filtering further supports map maintenance by identifying and removing unstable map elements associated with moving objects. In recent years, Semantic BKI has enhanced semantic consistency through spatially continuous probabilistic optimization, while ConceptFusion, combined with vision-language models such as CLIP, has driven semantic representations toward open vocabularies and high-dimensional feature spaces.

#### 4.1.4. 3D Gaussian Map Update Strategies

Kerbl et al. first proposed the Adaptive Density Control update scheme, establishing the first dynamic map update mechanism for 3D Gaussian Splatting (3DGS) [48]. During optimization, the system continuously monitors the view-space gradients of each Gaussian primitive: it performs cloning or splitting for under-reconstructed regions, while pruning primitives with excessively low opacity or excessively large scale. This work formed the first gradient-guided Gaussian map addition/deletion update framework, laying the foundation for subsequent online Gaussian map construction.

Building on this work, Keetha et al. proposed the Silhouette-Guided Incremental update scheme for SplaTAM [50]. Using streaming RGB-D data, the system renders the current viewpoint silhouettes in real time, and instantiates new Gaussian primitives only in unmapped blank regions. For already covered areas, it only optimizes existing Gaussian parameters, thus avoiding redundant Gaussian generation and enabling continuous incremental growth of online dense maps.

Yan et al. further proposed the Uncertainty-Guided Expansion update scheme for GS-SLAM [49]. Unlike methods that rely on depth residuals to expand maps, this method takes rendering uncertainty as the criterion for adding new Gaussians, generating new Gaussian primitives only at positions where current Gaussians cannot adequately explain observations. Combined with joint camera tracking and Gaussian-map optimization, this strategy improves the accuracy and stability of online map updates.

As the scale of Gaussian maps continues to expand, research has begun to focus on structural optimization and memory efficiency during the update process. Lu et al. proposed the Anchor-based Neural Growth update scheme for Scaffold-GS, which uses sparse voxel anchors instead of directly maintaining a large number of Gaussian primitives [208]. It dynamically decodes local Gaussians based on the current viewpoint via MLPs, achieving structured growth, effectively suppressing floating artifacts and significantly reducing memory consumption.

To address the problem of Gaussian shape degradation caused by the lack of depth constraints in monocular SLAM, Matsuki et al. proposed the Keyframe-Based Regularized update scheme for MonoGS [74]. The system jointly optimizes the map within a local keyframe sliding window, and introduces isotropic regularization to constrain newly generated Gaussians to maintain approximately spherical shapes, avoiding excessive stretching and improving geometric stability during incremental updating.

Targeting city-level ultra-large-scale scenes, Lin et al. proposed the Progressive Data Partitioning update scheme for VastGaussian [239]. This method divides the entire space into multiple independent data chunks, dynamically loads local regions according to camera visibility, performs gradient updates only on the current data chunk, and finally completes global seamless stitching. It thus breaks through the single-GPU memory limitation and realizes efficient local updating of ultra-large-scale Gaussian maps.

Meanwhile, Gaussian map updating has expanded from static scenes to dynamic environments. Luiten et al. proposed the Persistent Physical State update scheme for Dynamic 3D Gaussians [73]. Abandoning the traditional “delete–rebuild” strategy, it continuously updates the positions and poses of existing Gaussians through 6-DoF rigid body transformations, enabling the map to directly represent object motion processes and realize the continuous evolution of physical states and map representations.

Furthermore, Wu et al. proposed the HexPlane Deformation update scheme for 4DGS, extending map updates from spatial dimensions to joint spatiotemporal modeling [240]. The system encodes deformation fields using multiple orthogonal 2D spatiotemporal feature planes. When new observations arrive, only the HexPlane feature parameters need to be updated, without frequent addition and removal of Gaussian primitives; it can drive base Gaussians to complete complex non-rigid deformations, achieving efficient four-dimensional dynamic map updating.

3D Gaussian map updating has gone through four stages: gradient-driven addition/deletion, online incremental updating, structured organization, and dynamic spatiotemporal evolution. Early methods achieved adaptive optimization through cloning, splitting and pruning of Gaussian primitives. Afterwards, combining silhouette masks and rendering uncertainty realized incremental generation of new regions and online optimization of existing parameters. In recent years, anchor decoding, spatial partitioning and dynamic modeling methods have driven map updating from single Gaussian optimization toward structured management and spatiotemporal evolution.

At present, 3D Gaussian maps have gradually formed an update paradigm of “local incremental growth + parameter optimization + structured management + global joint optimization”. By continuously adjusting the position, scale, color and opacity of Gaussians, combined with pose optimization, it realizes high-quality, scalable real-time mapping. In the future, it will further integrate multiple representations such as TSDF, Mesh, neural implicit fields and scene graphs, and combine semantic perception, continual learning and dynamic forgetting mechanisms to achieve long-term autonomous evolution, providing a unified, high-fidelity and sustainably updatable hybrid map foundation for embodied intelligence.

A cross-cutting question raised by the update strategies in Section 4.1 is how each representation copes with the two dominant failure modes of long-term operation: moving objects (which corrupt the static map) and accumulated pose error (which distorts it globally). Table 6 summarizes the two mechanisms per representation, revealing two convergent designs—temporal-persistence gating for dynamic objects, and pose/geometry decoupling for drift.

### 4.2. Submap-Based Hybrid Map Update and Management

Submap-based hybrid maps have become the dominant framework for long-term map management because they embody a single organizing principle: once a local submap is sufficiently stable, it is frozen, and all subsequent global corrections are confined to a higher-level pose graph. This decouples real-time local updating from asynchronous global consistency maintenance—a decoupling that is the real object of study in this line of work, regardless of the underlying data structure.

**Spatial submap partitioning.** The paradigm originates in spatial submap partitioning. Stückler and Behnke [241] maintain multi-resolution surfel submaps and freezes one whenever its pose uncertainty exceeds a threshold, so that global consistency is achieved only through constraints between historical submaps. Fioraio et al. [242] divide space into overlapping TSDF subvolumes and folds their inter-subvolume registration errors into a global bundle adjustment, correcting subvolume poses rather than the underlying voxels. RTAB-Map [243] applies the same idea at the topology level, binding submaps to graph nodes so that only node poses are ever updated. Together these systems establish the core rule: correct poses, not geometry.

**Object-level submaps.** The partitioning principle then extended from space to semantics. MID-Fusion [244] separates dynamic objects from the static background and maintains independent object submaps that evolve under their own motion models while the background retains global consistency. The dynamic/static distinction itself becomes a submap boundary, so that a moving object no longer contaminates the static map.

**Neural implicit submaps.** With neural implicit representation, the submap unit changed from voxels to learnable parameters. DI-Fusion [245] replaces TSDF with local MLPs, freezes each neural submap when the robot leaves its area, and emits a local mesh on demand. This preserves the freeze-and-optimize discipline while reducing storage overhead, marking the transition from voxel updating to neural parameter updating.

**Multi-layer and neural hybrid submaps.** The most recent systems update several layers jointly within the same submap framework. S-Graphs+ [82] maintains points, planar structures, and topology in one factor graph, so that a loop closure updates all layers synchronously. SLAMesh [34] moves mesh generation into the front-end time window. PIN-SLAM [51] organizes the map as sparse neural implicit submaps and, at global optimization, adjusts only inter-submap poses without retraining or re-fusing point clouds—completing the decoupling of neural representation updating from global geometric correction.

The paradigm that has emerged—local incremental updating, submap freezing, global pose optimization, and hybrid recombination—is valuable precisely because of the decoupling it achieves: real-time local updates, bounded global computation, and coordinated maintenance across geometry, semantics, topology, and neural fields. It has therefore become the dominant technical route for long-term mapping and map management in single-robot systems.

### 4.3. Hardware–Software Co-Design and Edge Deployment Trade-Offs

The map representations reviewed in Section 2 and Section 3 differ not only in accuracy and expressiveness but also in their deployability on real embodied platforms. A single robot is typically constrained by an onboard compute budget (tens of TFLOPs for an edge GPU), a few gigabytes of memory, a power envelope of a few watts to tens of watts, and thermal limits that throttle sustained computation. Classical explicit representations—occupancy grids, octrees, and distance fields—are the most deployment-friendly: their updates are incremental and they run on CPUs (e.g., OctoMap clamps and prunes octree nodes [16]; Voxblox computes ESDF incrementally on CPU [17]). Neural implicit maps invert this trade-off: they achieve a high compression ratio but pay for it in expensive online optimization (NICE-SLAM [44] is seconds-per-frame), while 3DGS recovers 30–100 fps rendering [48] at the cost of 10^5^–10^6^ Gaussian primitives and a hard dependency on GPU rasterization. A growing line of work targets lightweight deployment through quantization and compression (quantized neural SLAM [187]; feature-plane/codec-based 3DGS compression [202] and compact 3DGS SLAM [124]), hardware–software co-design (OctoCache caching for 3D occupancy [138]; SuperNoVA resource-aware SLAM [155]), and streaming/partitioning (VastGaussian chunking [239]; PIN-SLAM submap freezing [51]). For the practitioner, the practical guidance is: (1) prefer explicit geometric maps for navigation under a CPU/low-power budget; (2) adopt 3DGS only when photorealistic rendering is required and an edge GPU is available, applying quantization from the outset; (3) reserve neural implicit maps for offline or compression-driven long-term scenarios. Hardware–software co-design, not algorithmic accuracy alone, will become a first-class design axis for embodied map systems.

### 4.4. Concluding Remarks on Map Update and Management

This Section systematically reviews the update mechanisms and evolutionary trajectories of diverse map representations for long-term autonomous operation of single robots. From classic map paradigms including 2D grid maps, point cloud maps, semantic maps, and 3D Gaussian maps to the widely adopted submap-based hybrid map update frameworks in recent years, various map update approaches have progressively transcended the limitations of traditional static mapping, gradually forming mature map lifecycle management systems tailored for long-term autonomous operation and dynamic environment adaptation.

Overall, although update strategies for different map representations are designed for distinct data structures, their evolutionary paths share remarkable internal consistency. Early research primarily relied on Bayesian updating, temporal decay, and probabilistic filtering to iteratively refine map states. Subsequently, decoupled maintenance of dynamic objects and static environments was realized through change detection, object-level management, semantic fusion, and high-efficiency spatial data structures, which enhanced the stability and reliability of maps in complex dynamic scenarios. In recent years, driven by advances in deep learning, neural implicit representations, and 3D Gaussian Splatting, map updating has gradually evolved from conventional data fusion to parameter optimization, incremental feature learning, and multimodal semantic fusion, enabling maps to simultaneously preserve geometric accuracy, semantic consistency, and long-term maintainability.

Meanwhile, map management paradigms have also gradually shifted from global holistic reconstruction to local incremental maintenance. Submap-based hybrid map updating achieves effective decoupling between local map updating and global consistency maintenance via historical submap freezing, local incremental optimization, and global pose graph adjustment. This framework markedly reduces computational overhead and storage burden in long-term operation, and further supports the coordinated maintenance of multi-modal map representations spanning geometry, semantics, topology, and neural implicit fields, thus becoming the dominant technical route for long-term mapping and map management in single-robot systems.

In general, single-robot map updating technologies have undergone a twofold evolution: from observation fusion-driven to continual learning-driven, and from single-map independent maintenance to hybrid-map collaborative management. Looking ahead, propelled by advances in embodied intelligence, vision-language models, world models, and continual learning, map updating will further evolve toward open-world adaptation, multimodal fusion, task-driven orchestration, and lifelong autonomous maintenance. This will enable maps to transform from static geometric descriptions of the environment into long-term spatial memory, knowledge organization, and intelligent reasoning platforms, delivering more unified, efficient, and sustainable map support for robots to perform persistent perception, planning, interaction, and autonomous decision-making in complex open environments.

## 5. Conclusions and Outlook

### 5.1. Conclusions

This paper has presented a systematic survey of map construction and management for single embodied agents in long-term autonomous operation, covering three dimensions: map representation, map construction, and map management and updating. First, in terms of map representation, we analyzed the evolutionary process of 2D occupancy grid maps, 3D voxel maps, distance field maps, feature maps, mesh maps, topological maps, semantic maps, neural implicit maps, 3D Gaussian Splatting (3DGS) maps, and hybrid maps. Research demonstrates that robot maps have gradually evolved from early geometric representations oriented toward localization and navigation to multi-level spatial representation systems integrating geometric structures, semantic knowledge, visual information, and task experience. Different map representations exhibit clear complementary relationships in accuracy, computational efficiency, semantic expressiveness, and task adaptability; a single map structure can hardly satisfy the multi-task, multi-scale, and long-term operation requirements of future embodied intelligent systems. Therefore, hybrid maps that fuse multiple representation forms have become an important development direction for current robot environment modeling.

Second, in terms of map construction, this paper summarized the construction methods corresponding to different map representations. From traditional probabilistic mapping, geometric registration, graph optimization, and factor graph optimization to recent online optimization methods based on semantic fusion, neural implicit representation, and 3DGS, map construction technology has gradually transformed from pure geometric reconstruction to comprehensive environmental modeling integrating spatial structure, visual information, and semantic knowledge. As embodied intelligence application scenarios expand from structured environments to open worlds, future map construction needs not only to improve geometric accuracy but also to address issues of dynamic environment adaptation, multi-sensor fusion, large-scale scene extension, and long-term consistency maintenance.

Finally, in terms of map management and updating, this paper summarized key technologies such as incremental updating, multi-session fusion, map compression, change detection, and neural map continual learning in the process of single-agent long-term operation. Traditional map updating mainly relies on new sensor observations for probabilistic fusion, while future embodied intelligence systems need to further achieve map lifecycle management, endowing maps with environmental change perception, historical information maintenance, knowledge accumulation, and active updating capabilities. Therefore, map management is gradually transforming from a traditional data fusion problem into a world model maintenance problem oriented toward long-term autonomous operation. 

What is established by the literature surveyed here can be summarized as follows: (1) maps have evolved from 2D grids to multi-layer hybrid representations, and hybrid architectures now dominate long-term operation because they decouple local updates from global consistency [69,81,82]; (2) moving-object handling and drift correction converge on the same two designs—temporal persistence gates and pose/geometry decoupling; and (3) neural and 3DGS maps achieve state-of-the-art fidelity but remain constrained by update latency and memory on embedded platforms. Beyond these literature-supported findings, we anticipate—but do not claim to prove—that task-driven abstraction, quantization-aware online mapping, and hardware–software co-design will become the dominant design axes for embodied map systems, and that maps will evolve from static geometric descriptions into persistent world models. These projections are our own, and their validation requires the open problems in Section 5.2 to be addressed.

### 5.2. Outlook

The field is converging on a redefinition of what a map is. Where maps were once static geometric records built for localization and planning, they are becoming task-aware, continually maintained models of the world able to persist across sessions, adapt to change, and answer queries at whatever level of detail a task demands.

**From single representations to a hierarchical world model**. Robot maps will evolve from single spatial representations toward hierarchical multimodal world models that integrate geometry, appearance, semantics, affordance, temporal dynamics, and task memory. At the base, a metric layer provides localization, scale recovery, and traversable-space modeling. Above it, a high-fidelity visual layer—built from point clouds, meshes, TSDF, NeRF, or 3DGS—delivers photorealistic reconstruction; 3DGS, with its high-quality rendering, efficient optimization, and scene editing, is the most promising candidate here, though it is unlikely to operate alone. Higher layers add open-vocabulary semantics and scene topology, then object affordances and physical interaction constraints, then spatiotemporal dynamics and state prediction, and finally runtime memory and experience management, so that each layer forms a closed cognitive loop through continuous updating.

**From geometric reconstruction to task-driven, learning-based construction**. Map construction will shift from pure geometric reconstruction toward cognitive modeling oriented to task understanding. On the one hand, SLAM will keep improving in precision and robustness through multi-sensor fusion and multimodal foundation models; on the other hand, construction will become increasingly learning-driven, using vision-language models, large language models, and world-model priors to predict unknown space, complete structure, and infer semantics. Construction targets will also shift from voxels, points, and meshes to learnable parameters such as neural features, implicit-field parameters, and Gaussian primitives, evolving from perceptual fusion, through geometric reconstruction and semantic understanding, toward world-model learning.

**From passive fusion to active, lifelong world maintenance**. Map management will transform from passive data fusion to active world maintenance. Long-term operation requires distinguishing permanent structural change from temporary dynamics and deciding, based on task requirements, what to update, retain, or forget. As operation time grows, continual-learning mechanisms—map compression, neural submap management, version control, memory replay, and catastrophic-forgetting suppression—become essential, and maps will extend from 2D/3D space to 4D spatiotemporal models that predict future states. Map updating thus evolves from probabilistic fusion, through dynamic and semantic maintenance, toward world-model continual learning.

**From single robots to multi-robot and cloud-edge collaboration**. As robots scale from single devices to fleets, map systems will extend toward multi-robot collaboration and cloud-edge integration, sharing local maps, object knowledge, and task experience. This raises open challenges in coordinate unification, data consistency, communication efficiency, and privacy. Future systems will combine distributed optimization, federated learning, and cloud-edge architectures, so that robot-side real-time perception and local updating coexist with cloud-side long-term knowledge maintenance, ultimately forming shared world models that connect perception, understanding, planning, action, and experience accumulation.

These trends raise a set of open research problems that, in our view, will define the next phase of the field.

**Cross-representation consistency for hybrid maps**. Hybrid maps stack geometric, semantic, topological, and neural layers, but today each layer is updated largely independently. When a loop closure or a detected change occurs, the correction must propagate to every layer without contradiction, yet no existing formulation provides a joint objective or consistency guarantee across layers. The open problem is how to update all layers jointly—for example through a shared factor graph or a synchronized parameterization—and to define, with a theoretical guarantee, what cross-layer consistency means under partial observation.

**Quantization-aware, online 3DGS for edge platforms.** 3DGS delivers photorealistic rendering but stores 10^5^–10^6^ Gaussians, and existing compression methods [124,202] are largely offline and post hoc. Real robots require compression that is quantization-aware, incremental, and budget-constrained. The open problem is how to quantize, prune, and update a 3DGS map online within a fixed memory and energy budget without catastrophic drift—including which Gaussians to retain by saliency or view coverage and how to retrain on the fly.

**Joint estimation of dynamic objects and long-term drift**. Dynamic-object removal and drift correction are currently solved as separate, sequential stages, yet they are deeply coupled: a misclassified moving object corrupts the static map and biases pose estimation, while residual drift makes it harder to distinguish what moved from where the robot is. The open problem is a single probabilistic estimator that jointly reasons about scene dynamics and sensor pose, resolving both in one model instead of through an error-prone hand-off boundary.

**Benchmarks for continual map learning and forgetting**. There is no standard metric or benchmark for catastrophic forgetting in robot maps; existing SLAM benchmarks measure one-shot accuracy rather than the stability of a map across months of appearance change, object turnover, and incremental updating. A task suite measuring memory stability, update cost, and forgetting over long simulated horizons would make progress quantifiable. More broadly, trajectory error, rendering quality, semantic accuracy, and task success each measure a different layer, and a unified metric of how much spatial intelligence a map actually contributes is still missing.

**Affordance- and task-driven map construction**. Current maps are built to a fixed, geometry-complete resolution regardless of what the robot will actually do. The open problem is task-conditioned construction: building the map only to the level of detail a task requires—a manipulable region versus a passable corridor—and letting natural-language goals actively select where to observe next. This requires connecting affordance prediction, active perception, and language grounding so that the map functions as a workspace rather than a static reconstruction.

**Privacy- and communication-aware multi-robot map fusion.** Extending single-agent maps to cloud-edge collaboration raises coordinate unification, data consistency, and privacy preservation under bandwidth constraints. Beyond standard distributed optimization, the open problems are which information to share—features, subgraphs, or learned latents—how to reconcile conflicts without a trusted central server, and how to federate learning so that raw sensor data never leaves the robot.

**Open-vocabulary, cross-session object identity.** Open-set recognition makes maps more queryable, but it simultaneously makes the core question “is this the same object as before?” harder to answer across viewpoints, lighting, and sessions. The open problem is how to keep object identities, coordinates, and interaction histories consistent across re-localization, map re-optimization, and software updates, when appearance-based similarity is unreliable and near-duplicate instances coexist. This calls for identity anchored in geometry and topology rather than appearance alone.

**Query-time map resolution and task-conditioned actionable memory**. Rather than fixing every object instance and semantic granularity at mapping time, a map may store only retrievable evidence and defer parsing until a task query arrives. This challenges the “illusion of the complete map”: real environments change continuously and future tasks are unknown, so exhaustive upfront parsing is both wasteful and short-lived. The open problem is how to decide, at query time, what to remember, what to resolve, and what to forget under a bounded evidence budget, so the map remains a task-driven workspace rather than an ever-growing complete map.

## Figures and Tables

**Table 1:** Comparison of this survey with existing surveys on spatial representation, SLAM, and mapping. (✔/○/— denote full/partial/no coverage.).

Survey	Primary Focus	Representation	Construction	Management	Single-agent Long-Term	3DGS and Foundation
Cadena et al. [3]	Visual SLAM robustness (front/back-end, loop closure)	○	✔	○	—	—
Campos et al. [4]	Visual/visual–inertial/multi-map SLAM	○	✔	○	○	—
Rosen et al. [5]	Inference and representation for SLAM	✔	✔	○	—	—
Deng et al. [6]	Best 3D scene representation (geometric → foundation)	✔	○	—	—	✔
Tosi et al. [7]	NeRF and 3DGS SLAM	✔	✔	○	—	✔
Chen et al. [8]	Semantic lifecycle via foundation models	○	○	✔	○	✔
Tan et al. [9]	World models for VLA agents	○	—	○	—	✔
Ours	Single-agent map repr. + construction + management (full lifecycle)	✔	✔	✔	✔	✔

**Table 2:** Three-category taxonomy of map representations and the defining criterion of each category.

Category	Defining Criterion	Sub-Types (Sections)	Representative Methods
Geometric/Metric maps	Explicitly encode spatial geometry, distances and occupancy in discretized or continuous coordinates, without semantic labels	2D occupancy grid (Section 2.1), 3D voxel (Section 2.2), distance field (Section 2.3), feature/surfel (Section 2.4), mesh (Section 2.5)	OctoMap, Voxblox, ElasticFusion, Marching Cubes
Semantic/Structural maps	Explicitly encode object identity, category, or spatial relationships (topology) beyond raw geometry	Topological (Section 2.6), semantic (Section 2.7)	SSH, Kimera-Semantics, 3D DSG, ConceptFusion
Neural/Foundation-model maps	Represent the environment through learnable parameters (network weights/Gaussian primitives) optimized from data, optionally with open-vocabulary/foundation priors	Neural implicit (Section 2.8), 3DGS (Section 2.9), hybrid (Section 2.10)	NICE-SLAM, 3DGS, GS-SLAM, S-Graphs+

**Table 3:** Hybrid map architecture types.

Hybrid Map Architecture Types	Core Data Flow Components	Analysis of Technological Advantages and Complementary Characteristics	Typical Applications of Embodied Intelligence
Topometric map	Local high-precision geometric grid/local point cloud + global graph-theoretic topological nodes and connected edges	It significantly mitigates the global geometric distortion induced by cumulative drift in large-scale long-range exploration; while preserving ultra-high local collision avoidance precision, it achieves millisecond-level computational efficiency for cross-regional path planning.	Long-range navigation for large-scale indoor warehousing and logistics robots and shopping mall service robots.
Hybrid sparse–dense surface	Sparse visual landmarks/line and plane feature points + continuous surface surfel field (Surfels/Mesh)	It thoroughly decouples the computational resource contention between high-frequency camera tracking and low-frequency mapping, ensuring stable ego-localization; for high-level operations, it provides an ultra-smooth rigid-body geometric interface that enables contact friction simulation.	Mobile manipulation tasks with dexterous manipulators, large-scale autonomous driving and outdoor 3D mapping
Hybrid 2–2.5D Elevation Representation	Underlying 2D occupancy grid + overlying Digital Elevation Model (DEM) + friction/semantic layer	Under the premise of memory-constrained on-board edge computing, it fully addresses safe travers ability assessment for uneven terrain; it extracts slope normals and surface roughness to avoid physical instability.	All-terrain wheel-tracked exploration rovers, field rescue operations and dynamic foothold planning for quadruped robots.
Hybrid Object-Relational Linguistic Graph	Instance-level point cloud clusters/3D bounding boxes + open-vocabulary features (CLIP) + LLM-inferred relation edges	It completely removes the constraints of fixed label vocabularies and supports abstract logical reasoning based on human natural language common sense (e.g., “find a container that can hold water”).	Home-assisted rehabilitation robots, dynamic object search in open worlds, and natural language human–robot collaboration
Hybrid Explicit-Implicit Neural Rendering Representation	Explicit sparse tracking points/spatial indexing via voxel grids + implicit Neural Radiance Field (NeRF)/3D Gaussian Splatting (3DGS)	It leverages explicit spatial grid indexing to guide and accelerate implicit ray query efficiency; it supports photorealistic differentiable rendering pipelines and can be readily integrated with novel view synthesis for self-supervised loop closure learning.	High-fidelity reconstruction of environments with complex multi-material objects (e.g., transparent glass, highly reflective metal), and digital twins for VR teleoperation

**Table 4:** Comparison of Common Map Representations.

Representation Type	Typical Data Structure	Advantages	Limitations	Typical Construction Methods	Typical Application Scenarios
2D Grid Map	2D array, probabilistic occupancy grid	Simple implementation; high planning efficiency; strong interpretability	Cannot represent elevation information; difficult to adapt to complex 3D environments	Ray casting, recursive Bayesian update, 2D occupancy grid SLAM [10,11]	Indoor mobile navigation, global path planning
3D Voxel Map	Voxel grid, octree, spatial hashing	Explicit representation of occupied/free space; supports 3D collision detection.	High memory consumption; complex dynamic updates	OctoMap, TSDF fusion, voxel hashing, Voxblox [16,17,21]	UAV navigation, manipulator obstacle avoidance, 3D environment perception
Distance Field Map (TSDF/ESDF)	TSDF, ESDF, voxel hashing	Continuous distance representation; suitable for trajectory optimization; provides collision gradients	Complex ESDF maintenance; high computational overhead	TSDF integration, ESDF wavefront propagation, Voxblox [17,21,55]	Trajectory optimization, real-time obstacle avoidance, mobile manipulation
Feature Map	Point cloud, surfel, keyframe	Rich geometric details; high localization accuracy	Rapid growth of data volume; dynamic points degrade registration performance	ICP registration, surfel fusion, ElasticFusion [25,56,57,58,59]	High-precision localization, local mapping, robotic grasping
Mesh Map	Triangular mesh, mesh topology	Continuous surface; fine geometric representation; suitable for physical interaction	Complex online updates; difficult topology maintenance	Marching Cubes, mesh reconstruction, elevation mapping [31,60,62,63]	Digital twins, contact planning, environment reconstruction
Topological Map	Graph structure (nodes and edges)	Compact storage; high global planning efficiency	Insufficient geometric information; limited local localization capability	Topological abstraction, topological SLAM, graph-based representation [36,70]	Long-range navigation, environment exploration, task planning
Semantic Map	Semantic voxels, instance graph, scene graph	Supports object-level understanding; strong semantic query capability	Complex data association; significant impact of semantic noise	Semantic segmentation, multi-view fusion, Kimera-Semantics [32,69,71]	Object search, grasping, human–robot interaction
Neural Implicit Map	MLP, NeRF, SDF, hierarchical feature grid	Continuous high-fidelity representation; high compression ratio	Slow online optimization; difficult continuous updates	NeRF, NICE-SLAM, neural implicit mapping [44,45,71,72]	Long-term mapping, environment reconstruction, differentiable rendering
3D Gaussian Splatting Map	Gaussian primitives, Gaussian set	Fast rendering speed; high reconstruction quality; supports real-time SLAM	Rapid growth of Gaussian count; complex dynamic maintenance	3D Gaussian Splatting, GS-SLAM, differentiable rasterization [48,49,50,73,75,76,77,78,79,80]	High-fidelity reconstruction, visual navigation, open-world perception
Hybrid Map	Submaps, multi-layer maps, hybrid data structures	Integrates geometric, semantic and topological advantages; balances accuracy and efficiency	Complex system architecture; difficult cross-representation maintenance	Kimera-Multi, multi-map collaboration, unified factor graph optimization [69,81,82]	Long-term autonomous operation, embodied intelligence, integrated navigation and manipulation

**Table 5:** Quantitative task-oriented comparison of map representations (memory overhead, update latency, geometric accuracy, computational cost, and adaptability to manipulation and language-guided navigation tasks).

Representation	Memory Overhead	Update Latency	Geometric Accuracy	Computational Cost	Manipulation	Language-Guided Navigation
2D occupancy grid	Low (KB–MB)	Low (ms, incremental Bayes)	Low–Med (2D only)	Low	Low	Low
3D voxel (OctoMap)	Med–High (octree-compressed)	Low–Med (ray casting)	Med	Med	Med	Low
Distance field (TSDF/ESDF)	Med (voxel + hash)	Med (wavefront)	Med–High (continuous)	Med–High	High	Low
Feature/point cloud/surfel	High (grows with points)	Med	High (detail)	Med–High	High	Low
Mesh	Med (topology compact)	High (topology rebuild)	High (continuous surface)	Med	High	Low
Topological	Low (graph, very compact)	Low–Med	Low (no metric detail)	Low	Low	Med
Semantic	Med–High (labels/objects)	Med	Med (segmentation-limited)	Med–High	Med	High
Neural implicit (NeRF/SDF)	Low–Med (high compression ratio)	High (online optimization ~s/frame)	High (continuous)	High (GPU training)	Med	Med
3DGS	High (10^5^–10^6^ Gaussians, hundreds MB)	Med (fast raster, incremental)	High (photoreal)	High (GPU)	Med–High	Med
Hybrid	High (multi-layer)	Med–High (cross-layer sync)	High	High	High	High

**Table 6:** Cross-cutting summary of how each map representation handles moving objects and accumulated drift over long-term operation.

Representation	Moving-Object Handling	Accumulated-Drift Handling
2D occupancy grid	HMM/FreMEn temporal modeling [222,223]; fading-memory decay [220]	Loop closure + pose graph; SLAM Toolbox lifelong map merging [14]
Point cloud/surfel	Removert (remove–revert) [233]; voxel background + spatio-temporal filtering [232]; NDT-OM erase [228]	ElasticFusion non-rigid deformation [56]; submap + pose graph
Semantic	Semantic-consistency-based surfel filtering (SuMa++) [225]; instance state machine (merge/spawn) [237]	Object-level data association [236]; factor-graph joint optimization [32]
3DGS	Dynamic 3D Gaussians persistent rigid-body update [73]; 4DGS HexPlane deformation [240]	GS-SLAM back-end pose graph [49]; keyframe sliding window [74]
Hybrid/submap	MID-Fusion separates dynamic objects from static background [244]	Submap freezing + pose graph (Voxgraph, PIN-SLAM) [51]

## Data Availability

No new data were created or analyzed in this study. Data sharing is not applicable to this article.
